# Brucellosis in cattle and buffalo in southern Italian provinces: trends in presence of territory-specific One Health measures

**DOI:** 10.3389/fmicb.2025.1609336

**Published:** 2025-06-06

**Authors:** Alessandra Mazzeo, Celestina Mascolo, Lucia Maiuro, Marco Esposito, Carlo Ferrara, Nicola Rossi, Vincenzo Di Chiro, Sebastiano Rosati, Elena Sorrentino

**Affiliations:** ^1^Department of Agricultural, Environmental and Food Sciences, University of Molise, Campobasso, Italy; ^2^Department of Prevention, Complex Structure Animal Health, Local Health Agency of Caserta, Caserta, Italy; ^3^Unit of Prevention and Veterinary Public Health, Naples, Italy; ^4^Department of Prevention, Complex Structure Animal Health, Regional Health Agency of the Molise Region (ASReM), Campobasso, Italy; ^5^Department of Agricultural, Forest and Food Sciences, University of Turin, Turin, Italy

**Keywords:** *Brucella*, buffalo brucellosis, cattle brucellosis, milk value chain, One Health, grazing practices, animal tracking, brucellosis self-monitoring

## Abstract

**Introduction:**

*Brucella* spp. infections in livestock continue to pose significant threats to public and animal health, as well as to local economies. In the European Union, *Brucella abortus*, which primarily affects cattle and buffaloes, has been successfully eradicated in some Member States and across northern and central Italy through mandatory eradication and control programmes. However, some provinces in southern Italy, including Caserta and Salerno (Campania Region), remain affected, while in other provinces, as in Isernia (Molise Region), brucellosis has temporarily re-emerged.

**Methods:**

The study examines brucellosis outbreaks occurring between 2019 and 2023 in these territories, focusing on livestock that play a key role in the milk value chain, an essential pillar for economic sustainability, environmental protection and cultural heritage preservation. Since brucellosis is a WOAH Listed Disease subject to mandatory notification, we analyzed data registered in Italian, European Union and International portals concerning these notifications. Furthermore, we correlated these data to the national and regional rules adopted in order to tackle bovine brucellosis, including the methods approved for the serological diagnosis of infection.

**Results:**

In Isernia, a mountainous area where cattle ranching and traditional herding are widespread and culturally significant, brucellosis re-emerged in the period 2019–2021, when 30 outbreaks arose, 50% of outbreaks were linked to grazing practices. Outbreaks were promptly extinguished through veterinary intervention due to the low density of farms, which typically house only a few animals, and new cases have not been detected since 2022. The provinces of Caserta and Salerno present a different scenario, as they are major hubs for water buffalo breeding. The stringent tailored control measures, implemented in these provinces through a regional programme, led to a gradual decline in the prevalence of buffalo brucellosis outbreaks in Caserta, that involved 8,766 heads in 2019 and 6,164 heads in 2023, and in the eradication in Salerno, with 369 positive heads in 2019 brought to 0 since 2022. The Caserta programme specifically addresses the province’s particular vulnerabilities, which include frequent flooding events and the presence of the largest and most densely concentrated water buffalo population in Italy.

**Discussion:**

The results highlight the fundamental importance of a One Health approach, which includes targeted interventions adapted to the specific context in which they are applied. This approach must actively involve key stakeholders, including farmers, researchers, public health authorities, and policymakers, and be supported by financial investments. Key components include strengthening biosecurity measures, implementing advanced animal traceability systems, continuous professional training programmes (including for farmers), expert-led information-sharing technologies, and promoting voluntary serological self-monitoring practices.

## 1 Introduction

Brucellosis is a zoonotic disease that has significant public health and economic impacts, particularly in middle-and low-income countries (Jamil et al., 2022). The recently estimated annual global incidence of human cases is 2.1 million, with Africa and Asia sustaining most of the global risk and cases, although areas within the Americas and Europe remain of concern (Laine et al., 2023).

Brucellosis affects several of the most important livestock species, including domestic species of the order *Artiodactyla*, suborder *Ruminantia*. Brucellosis in cattle and buffalo, referred to collectively as bovine brucellosis, is primarily caused by *Brucella abortus*, while in sheep and goats, it is mainly due to *B. melitensis* (Regulation (EU), 2016; Commission Implementing Regulation (CIR), 2018; World Organisation for Animal Health (WOAH), 2022).

*B. suis*, which typically infects pigs, has also been occasionally detected in cattle, sheep, goats, horses, camels, and dogs. In the European Union (EU), *B. suis* biovar 2, which is very rarely pathogenic to humans (EFSA, 2009), is widely distributed among wild boars and hares. These wildlife species serve as significant reservoirs and can transmit the infection to domestic livestock. Thus, wildlife poses a spillover and spillback infection risk, necessitating management (Jamil et al., 2022; Savini et al., 2023; Ebani et al., 2022; Montagnaro et al., 2020; De Massis et al., 2019; Parolini et al., 2024).

In cattle, brucellosis is primarily transmitted via direct contact with vaginal discharges, aborted fetuses, or birthing materials from infected animals. Transmission may also occur via direct or indirect contact with a contaminated environment, through mucous membranes or broken skin (Spickler, 2018; Robi et al., 2023). Shedding in milk, semen and urine occurs. Transplacental and breast-feeding vertical transmission are graphically summarized in Supplementary file S1 (S1 – Transplacental and Breast-Feeding Vertical Transmission in Cattle). *Brucella* cells can survive for up to 8 months outside the animal host at high humidity and without direct sunlight exposure. Promiscuity and sharing outdoor pastures can facilitate the spread of the infection.

*B. abortus*, *B. melitensis*, *B. suis* (serovars 1 and 3), and *B. canis* are the most common species responsible for human brucellosis. Humans can acquire the infection through direct or indirect contact, inhalation, or exposure of abraded skin with infected animals and contaminated environment, particularly via contact with aborted fetuses, placenta tissue, amniochorionic membranes, and lochia, or through consumption of unpasteurized dairy products, as well as ingestion of undercooked meat products (Casalinuovo et al., 2016; Dadar et al., 2019; Khurana et al., 2021; Santos et al., 2021; Wang et al., 2024).

**Figure 1:**
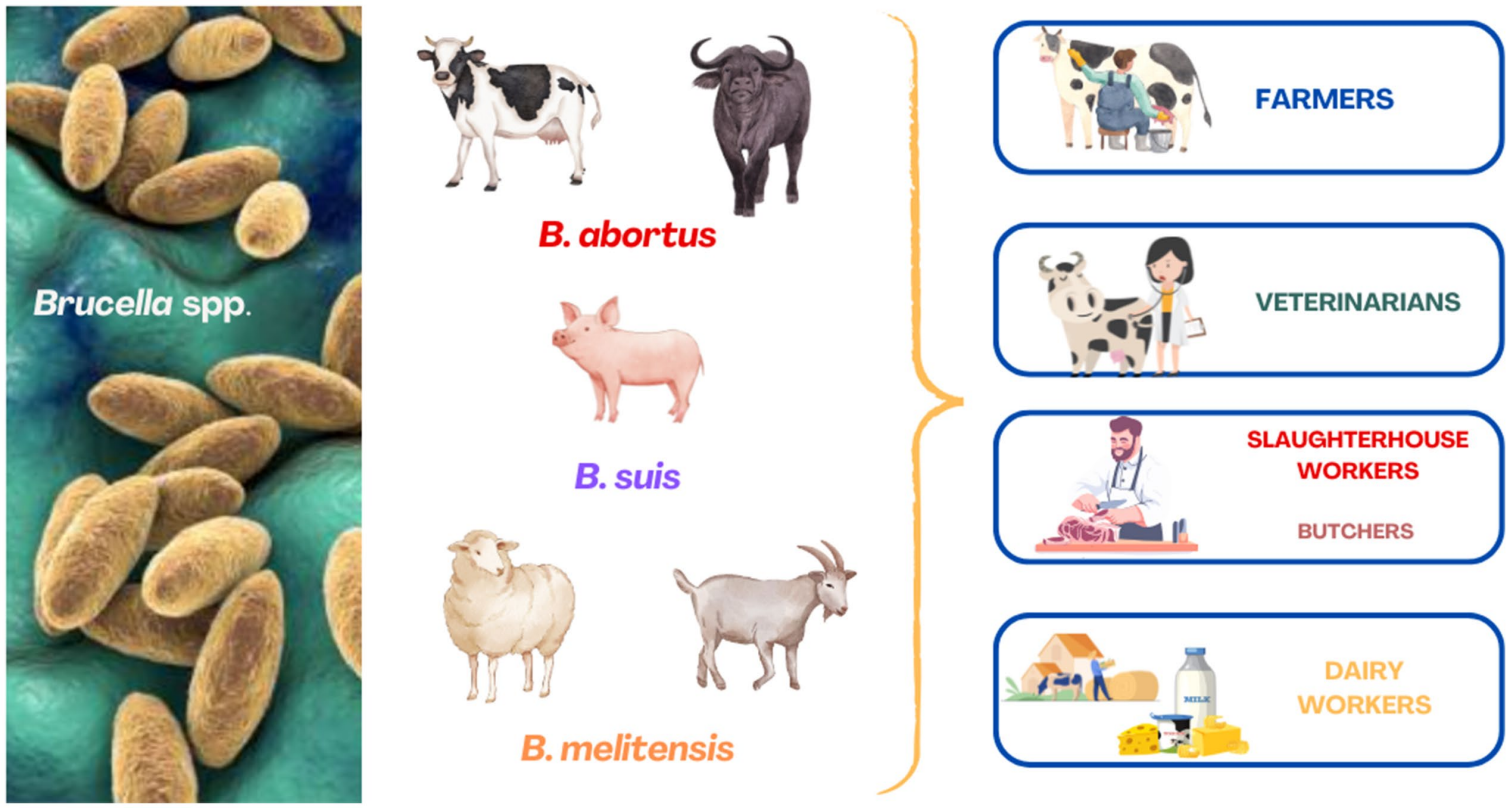
Occupational groups at elevated risk of contracting brucellosis from infected farm animals.

Human-to-human transmission is very rare and is mainly related to blood transfusion, bone marrow transplantation, sexual intercourse, and lactation (González-Espinoza et al., 2021). Brucellosis during pregnancy should be considered a significant risk factor for adverse pregnancy outcomes in humans (Arenas-Gamboa et al., 2016). The detection of a human brucellosis case in an area where the disease is absent in farmed animals, given the rarity of human-to-human transmission, raises immediate concern regarding the potential re-emergence of the infection in livestock or its presence in wildlife (Kamath et al., 2016; Lambert et al., 2021). Occupational groups at risk include farmers, veterinarians, dairy workers, butchers, and slaughterhouse workers (Figure 1) (Lounes et al., 2022; Mia et al., 2022; Dadar et al., 2023). According to the World Health Organization (WHO), brucellosis is classified among the top seven neglected zoonotic diseases.

Although strict control measures in animal husbandry have led to its eradication in many countries or zones, the disease remains present or suspected in others (WOAH-WAHIS).

In the European Union (EU), *Brucella abortus* infection has been eradicated in numerous Member States through mandatory eradication and control programmes, which have been progressively updated until the new European Union animal health law, the Regulation (EU) 2016/429 (Regulation (EU), 2016), and related complementary rules that establish norms for the management and prevention of animal diseases, including zoonoses. In areas that acquired the disease-free status (DFS), annual mandatory surveillance programmes are conducted to confirm their DFS, while targeted eradication programmes are implemented in non-DFS areas (Commission Implementing Regulation (CIR), 2024).

Italy participates in the EU mandatory eradication and surveillance programmes for brucellosis in cattle and buffalo, which have successfully led to the achievement of DFS in most Regions of northern and central Italy, as well as in certain provinces in the south. The Italian mandatory eradication and control programme for *B. abortus, B. melitensis*, and *B. suis* infections in bovine animals for the years 2024–2030 aims to maintain or achieve the DFS in Provinces, Regions and then in the whole Italian territory through a test-and-cull strategy. It also mandates measures for the eradication or surveillance of bovine (*Bos taurus*, *Bos indicus, Bison bison* and *Bubalus bubalis*) tuberculosis and brucellosis in sheep and goats (Italian Ministry of Health, 2024). The Decree seeks to establish and/or preserve DFS within the territory by guaranteeing 100% of scheduled inspections on establishments and animals, in compliance with EU regulations, with a planned annual reduction of at least 10% in disease incidence at the provincial level. Progress is assessed through the VETINFO information system. The programme aims for complete eradication of brucellosis and tuberculosis in Italy by 2030. The annually scheduled activities aim to obtain DFS through the implementation of surveillance based on:

- Inspection of establishments using official diagnostic methods and self-monitoring procedures.
- Surveillance of abortions.
- Slaughterhouse surveillance.
- Control of animal movements.

The programme mandates surveillance measures in DFS provinces and Regions (Supplementary file S2 – EU and Italian Regulatory Frameworks on Animal Health with a Focus on Grazing Practices).

Testing protocols are based on the health status of regions or provinces, with annual brucellosis testing for bovines aged 12 months and older. In designated cluster areas within non-free provinces, two annual tests are carried out in all DF bovine herds.

Official serological tests annually carried out in blood samples from all animals subject to the programme include Rose Bengal Test as screening test, Complement Fixation Test as confirmatory test, and Indirect-ELISA as a complementary test. Milk–ELISA can be used as a screening tool for pooled or bulk milk samples of unvaccinated DFS cattle herds located in DFS provinces, where at least 30% of lactating cows are in milk production. In these circumstances it can be used also in self-monitoring procedures but only if serological controls ensure that, while enabling the prompt detection of the presence of *Brucella* spp. infection, tests do not allow identification of positive animals. Confirmatory tests, in cases of positivity, must be conducted on serum samples collected from individual animals. If positivity arises in self-monitoring procedures, individual samples must be collected by official veterinarians and tested in official laboratories.

In cases of serological positivity, confirmatory testing via bacteriological and molecular methods (PCR or Whole Genome Sequencing) is required in disease-free establishments; otherwise, in previously infected herds, confirmatory tests are unnecessary since the case is considered epidemiologically connected to a confirmed case (Supplementary file S3 – Diagnostic Tests Adopted for Brucellosis in Italy under the Ministry of Health Decree of May 2, 2024, and Future Perspectives).

Stringent tailored measures are adopted in confirmed outbreaks. Surveillance is further supported by monitoring slaughtered animals, through *ante mortem* and *post mortem* inspection, with possible laboratory isolation of the causative agent from animals showing suspicious lesions. Furthermore, clinical cases (abortion, retained placenta, orchitis and arthritis) can lead to detection of the etiological agents in proper samples.

Outbreaks arise when the infection is detected, leading to the quick culling of positive animals. Additionally, milk from positive animals is excluded from human consumption, while milk from negative animals must be pasteurized and yield a negative result in the lactoperoxidase test. Strict biosecurity measures, such as isolating infected animals, disinfecting stables, and sanitizing pastures, must be implemented to prevent further spread of the infection.

In this study, we described the differences in brucellosis occurrence among cattle (*Bos taurus*) and water buffalo (*Bubalus bubalis*), mapped brucellosis spatial distribution and revealed temporal prevalence/incidence trends in three selected non-DF provinces in southern Italy: the province of Isernia in the Molise Region, and the provinces of Caserta and Salerno in the Campania Region (Italian Ministry of Health, 2024; Mazzeo et al., 2023). Furthermore, we discussed possible solutions to safeguard human and animal health and welfare, protect the environment, support the local economy, and preserve these territories cultural heritage, which may lead to disease decline in these areas.

## 2 Materials and methods

### 2.1 Selection of territories and period of observation

The neighboring or close non-DFS provinces selected in this study, due to their key role in the milk value chain and their marked differences in territorial and farming features, are:

- The province of Isernia, in the Molise Region, traditionally dedicated to cattle breeding, predominantly mountainous, and characterized by low farm density, small herd size, and grazing practices.
- The provinces of Caserta (bordering Isernia) and Salerno, both located in the Campania Region and largely characterized by flatlands crossed by rivers, provide favorable conditions for the traditional breeding of water buffaloes. Despite these advantageous conditions, these areas are subject to flooding and inundations, especially the province of Caserta, which was once a marshy area.
- In Caserta, grazing is prohibited in local jurisdictions identified as infection cluster municipalities, where the infection cluster areas (territories within a 2 km buffer around establishments with at least two active outbreaks in the past 2 years, one of which must have recurred in the last 5 years, or three active outbreaks in the last 2 years) cover more than 50% of the municipal extension (Campania Region, 2022), (Supplementary file S4 – Campania Region - Rules for Cluster Areas).

The observation period selected for analyzing the data on cattle brucellosis (Isernia) and buffalo brucellosis (Caserta and Salerno), and for assessing the effectiveness of the measures adopted to control the spread of the disease, covers the five-year period from 2019 to 2023. This timeframe was chosen to ensure the comparability of results across the entire five-year span, during which the mandatory bovine brucellosis eradication and control programme included only breeding herds. Starting in 2024, all bovine herds in Italy, both breeding and fattening, have been incorporated into the programme. Consequently, there is a lack of homogeneity in the results compared to previous years, due to the significant expansion of the total bovine population used to calculate positivity rates, incidence, and prevalence of infection.

Data on brucellosis from the European Union are also available only up to 2023. In the context of the national Italian framework, where data for 2024 were available, the three-year period from 2022 to 2024 was reported in the study.

### 2.2 Data collection

Brucellosis is a WOAH Listed Disease subject to mandatory notification. Bacteriological, molecular and serological tests are exclusively performed in authorized official laboratories. Data are then registered in Italian, European Union and International portals, that we used to collect them.

- VETINFO portal of the Italian Ministry of Health – developed by Istituto Zooprofilattico Sperimentale of Abruzzo and Molise (IZS AM) “Giuseppe Caporale” also hosting the National Reference Centre (CNR)^1^ for Brucellosis and the National Reference Laboratory (NRL) (see footnote 1) for Brucellosis – that serves as a comprehensive platform for collecting and managing veterinary and non-veterinary data critical for Italy’s Animal Health and Food Safety systems (VETINFO).^2^ It comprises also the following sections.
- SIR – Reporting Information System (Sistema Informativo Rendicontazioni), (SIR)^3^ that collects and transmits data to the Animal Disease Information System (ADIS), a European Commission (EC) platform.
- SIMAN – National Information System for Animal Disease Notification, that is a centralized platform for managing and analyzing data on animal disease outbreaks in Italy (SIMAN) (see footnote 2).
- The World Animal Health Information System of the World Organization of Animal Health (WOAH-WAHIS)^4^, the global animal health reference database of WOAH.
- European Union Food Safety Agency (EFSA) and European Union Centre for Disease Prevention and Control (ECDC), that share data, atlas, and reports accessible in open access.

### 2.3 Data elaboration

- Prevalence is the number of establishments with confirmed outbreaks during the period under investigation, expressed as a percentage of the total number of establishments tested. It was calculated using the formula (A/B) × 100, where:

A: number of establishments with a confirmed outbreak also in the previous year which reported at least one positive result during the year.

B: number of establishments tested (each establishment was counted only once, even if tested multiple times during the year).

- Incidence is the number of establishments with a newly confirmed outbreak in the year in question, following at least one positive result in a single animal, expressed as a percentage of the total number of establishments tested. It was calculated using the formula (C/B) × 100, where:

C: number of establishments with a newly confirmed outbreak in the reference year;

B: number of establishments tested (each establishment was counted only once, even if tested multiple times during the year).

- Maps to assess the spatial distribution of farms with outbreaks were created using QGIS Geographic Information System, version 3.24.0 Tisler (Free Software Foundation, Inc., Boston, MA 02110-1301 United States). The database included all registered brucellosis outbreaks in water buffalo reported in VETINFO. The data, including geographical coordinates, the identification number of the farm provided in the form of Excel datasheets. The shapefile of the study area was downloaded from the Gistat website (ISTAT, 2023). We imported the shapefile and Excel datasheets into the QGIS software and converted the Excel data into point data.
- The heatmaps, illustrating the distribution of spatial density of establishments with outbreaks in the provinces of Caserta and Salerno, were generated using Heatmap plugin. This tool uses kernel density estimation to interpolate a continuous density surface from a set of input points. To create the heat maps a search radius of 7 points (outbreaks) was applied, and the triweight kernel function was selected to assign greater weight to closer points (maximum value: 6.8).
- Geographical clustering analysis of the establishments with infected heads in the provinces of Isernia was performed using Python 3.8 (Python Software Foundation).
- To assess non-linear changes in the number of brucellosis-positive heads over time, a quadratic regression analysis was conducted using Microsoft Excel (16.94 version). A second-degree polynomial model was applied to annual data from 2017 to 2023. The statistical significance of the model was evaluated using a *p*-value threshold of 0.05.
- Positive cases from the provinces of Caserta and Salerno were analyzed using the Spatial Variations in Temporal Trends (SVTT) analysis using Python 3.8 (Python Software Foundation). This analysis was conducted to assess the spatial distribution and temporal trends of brucellosis outbreaks.

## 3 Results

### 3.1 Background

From 2019 to 2023, the number of new *B. abortus* outbreaks in cattle and buffaloes across the EU decreased significantly, from 485 positive herds in 2019 to 284 in 2023 (EFSA and ECDC, 2024). Extending our analysis to EU data concerning the year 2024 was possible by accessing the continuously updated World Animal Health Information System. In 2024, 343 outbreaks are reported, affecting 5,793 animals, of which 292 were culled and 5,040 slaughtered (WAHIS – WOAH).

**Figure 2:**
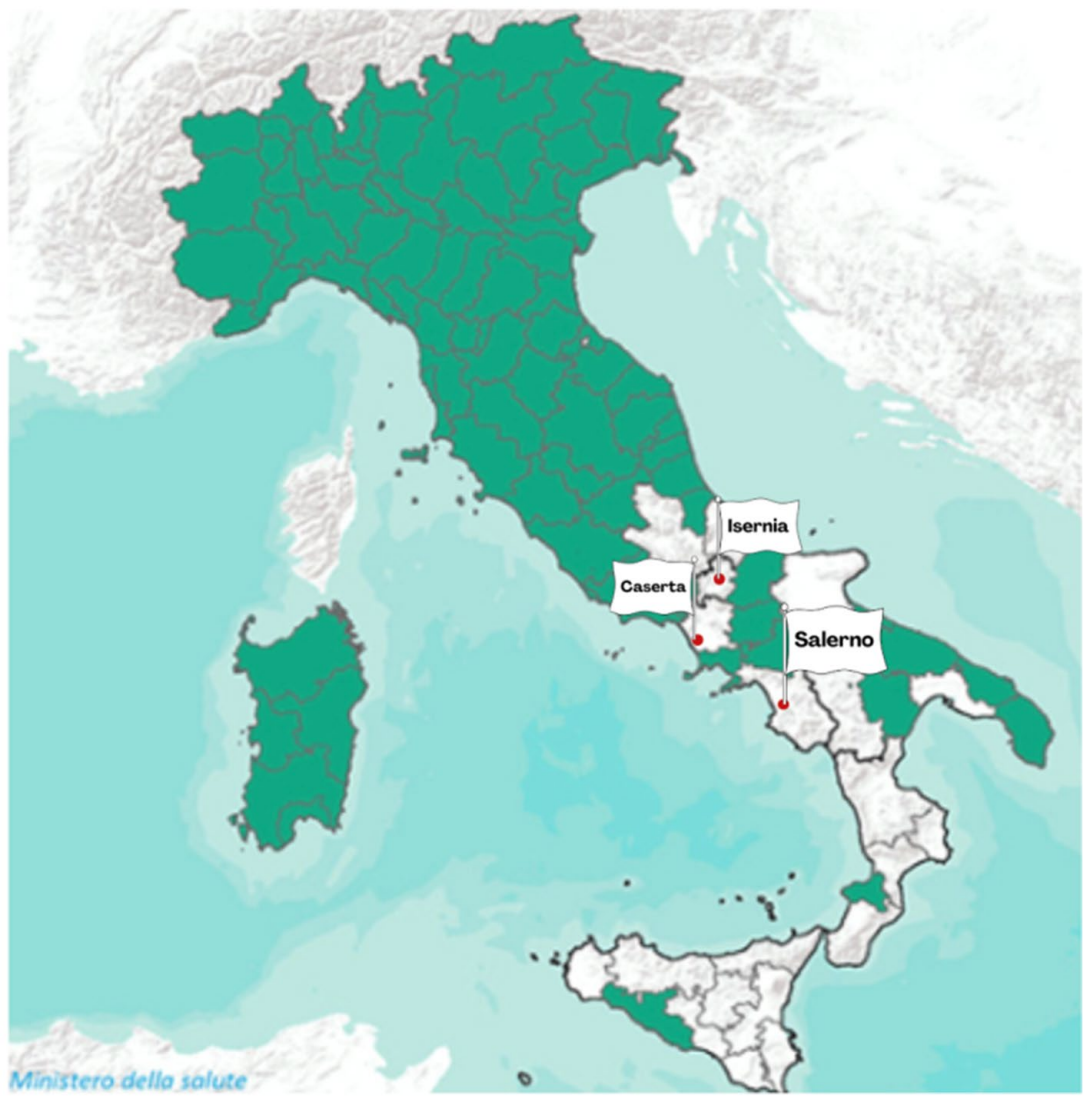
DFS (green areas) of Italian Regions and Provinces concerning bovine brucellosis (Commission Implementing Regulation (CIR), 2024, modified from VETINFO Portal of the Italian Ministry of Health).

In the EU, brucellosis remains a concern in some Member States. Bulgaria, Greece, Hungary, Italy, and Portugal are still working on eradication programmes, although Italy and Portugal have some zones with Disease-Free status (DFS) for bovine brucellosis (EFSA and ECDC, 2024). Italian Regions and Provinces that acquired the DFS are reported in the Commission Implementing Regulation (EU) 2024/1332 (Commission Implementing Regulation (CIR), 2024). In Figure 2, the DFS provinces are colored in green, and those that are not DFS are colored in white. The provinces of Isernia, Caserta, and Salerno, which are the object of this study, are highlighted with a red dot.

Extending our study through the consulted databanks, in the period 2022–2024, in Italy, a reduction in both cases and outbreaks of brucellosis in cattle and buffalo and in their prevalence and incidence was recorded (Table 1). In particular, confirmed outbreaks of brucellosis in cattle decreased from 163 in 2022 to 65 in 2024, with a reduction of approximately 60%. A similar trend is observed for confirmed outbreaks of brucellosis in buffaloes, which decreased from 56 in 2022 to 30 in 2024, with a reduction of approximately 47%.

**Table 1:** Cattle (*Bos taurus*) and buffalo (*Bubalus bubalis*) cases and outbreaks of brucellosis in Italy in the period 2022–2024.

Years	Cattle (*Bos taurus*)			Buffaloes (*Bubalus bubalis*)		
	2022	2023	2024	2022	2023	2024
Number of establishments with outbreaks	241	139	104	96	87	67
Prevalence*	0.19%	0.11%	0.09%	4.09%	3.82%	3.07%
Number of establishments with new outbreaks confirmed in the year	163	96	65	56	53	30
Incidence**	0.13%	0.08%	0.05%	2.39%	2.33%	1.37%

*Prevalence: Percentage of establishments that had a confirmed disease outbreak in the previous year and reported at least one positive result during the year in question, calculated over the total number of establishments tested. Each establishment is counted only once, even if inspected multiple times during the year. **Incidence: Percentage of establishments with a newly confirmed disease outbreak in the year in question, following at least one positive result in a single animal, calculated over the total number of establishments tested.

**Figure 3:**
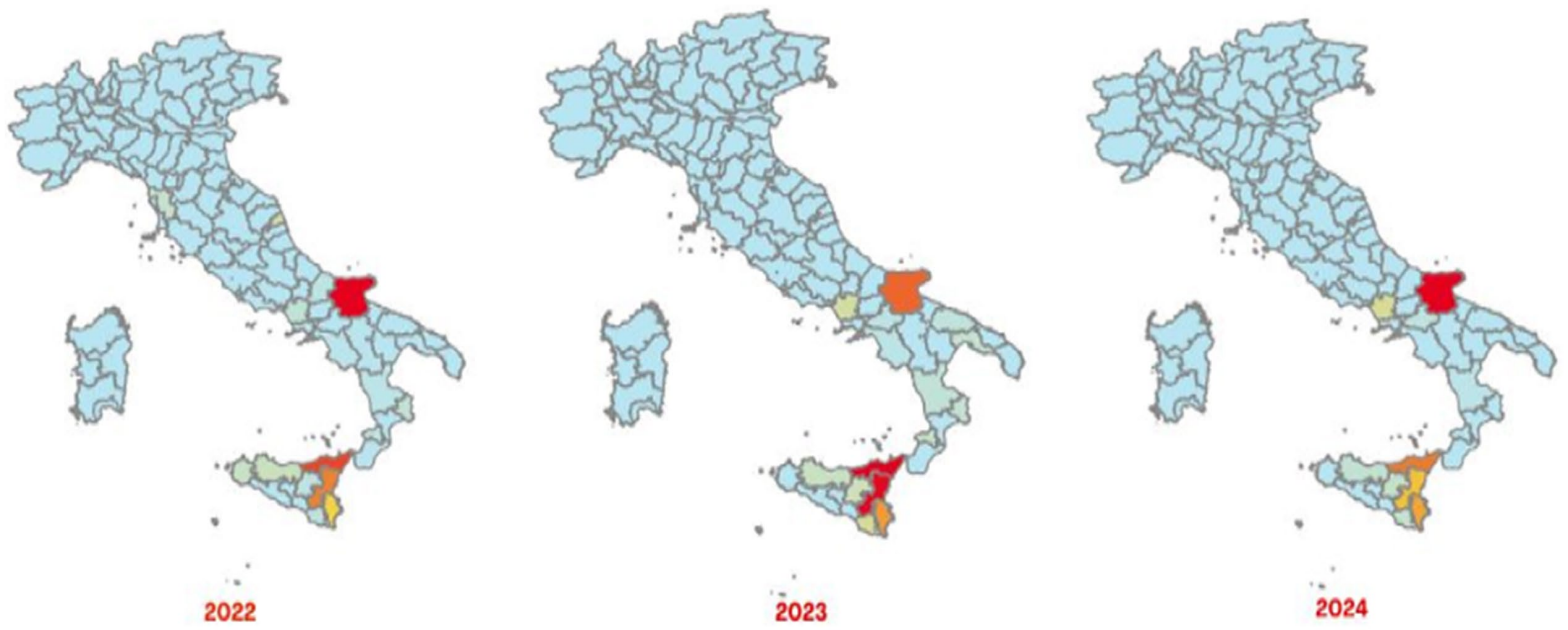
Maps of confirmed cases of cattle brucellosis recorded in Italy in 2022–2024 (red areas represent the highest number of cases and light green areas represent the lowest ones) (SIMAN).

**Figure 4:**
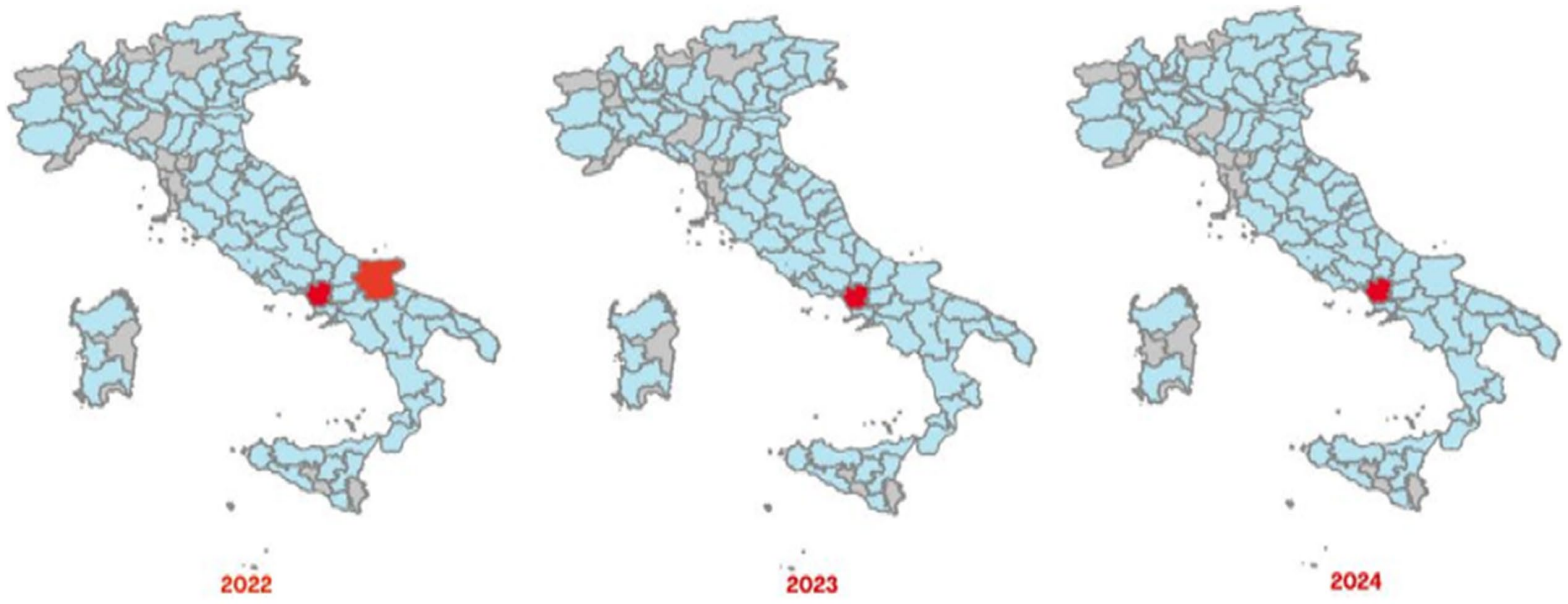
Maps of confirmed cases of buffalo brucellosis (red areas) recorded in Italy in 2022–2024 (SIMAN).

The Italian Regions most affected by brucellosis outbreaks in bovine animals, intended as cattle, buffaloes and other species indicated in the animal health law in force, are the southern ones, with a different geographical distribution between cattle brucellosis outbreaks (Figure 3) and buffalo brucellosis outbreaks (Figure 4).

In the period 2022–2024, the areas most affected by cattle brucellosis outbreaks were the province of Foggia, in Apulia Region, and several provinces of Sicily Region (Figure 3). In contrast, buffalo brucellosis outbreaks (Figure 4) were primarily recorded in the provinces of Caserta and Foggia in 2022, and subsequently only in the province of Caserta (one of selected areas selected in this study) in 2023 and 2024.

### 3.2 Results concerning the province of Isernia

Cattle farming in the province of Isernia, in 2023, comprised a total of 660 farms and 14,462 cattle heads, of which 389 breeding establishments housing 12,021 cattle heads included in the mandatory programme for brucellosis, that benefit from the rich Apennine pastures, which reach altitudes of up to 1,400 meters. The dairy production-oriented establishments host a small number of heads, in numerical decline in Table 2.

**Table 2:** Consistency classes of dairy production-oriented establishments in the province of Isernia in the period 2019–2023 (VETINFO).

Consistency classes	2019		2020		2021		2022		2023	
	No. of farms	No. of heads	No. of farms	No. of heads	No. of farms	No. of heads	No. of farms	No. of heads	No. of farms	No. of heads
0 Heads	3	0	2	0	8	0	7	0	7	0
1–2 Heads	10	17	7	10	9	13	7	9	6	10
3–5 Heads	13	47	11	42	11	47	17	67	13	56
6–9 Heads	18	130	19	141	20	153	18	136	12	89
10–19 Heads	46	676	38	521	37	523	33	489	35	494
20–49 Heads	59	1,888	60	1,834	54	1,746	50	1,590	47	1,481
50–99 Heads	29	2,003	26	1,743	26	1,870	27	1,913	27	1,935
100–499 Heads	11	1,583	13	1,867	11	1,659	11	1,647	8	1,364
>500 Heads	2	1,446	2	1,557	2	1,606	2	1,657	2	1,680
Total	191	7,790	178	7,715	178	7,617	172	7,508	157	7,109

Table 3 details the 13 outbreaks of cattle brucellosis, causing 8 stamping out operations, that suddenly re-emerged in that province in 2019 after that, in 2018, a single animal tested positive out on a total of 12,605 animals representing the 99.68% of 12,646 animals included in the eradication programme and hosted in 495 establishments (average number of cattle per farm: 25.5).

**Table 3:** Cattle brucellosis outbreaks occurred in the province of Isernia, in Molise Region, in the period 2019–2023 (since 2022 no outbreaks occurred).

Sequential numbers	Outbreak code	Farm latitude	Farm longitude	Type of farming	Origin of outbreak	Culled heads	Total heads
1	2019/400 – IS	41,63,386	14,44,321	Stationary	Unknown	52	52
2	2019/429 – IS	41,602,441	14,37,639	Transhumance	Unknown	116	129
3	2019/433 – IS	41,628,151	14,407,676	Extensive	**PASTURE**	58	58
4	2019/449 – IS	41,611,783	14,4,585	Stationary	Unknown	91	96
5	2019/450 – IS	41,6,118	14,458,483	Extensive	Unknown	87	96
6	2019/451 – IS	41,5,945	14,310,317	Stationary	Unknown	3	31
7	2019/455 – IS	41,610,217	14,4,524	Stationary	Unknown	3	60
8	2019/456 – IS	41,5,865	14,342,867	Stationary	Unknown	3	24
9	2019/457 – IS	41,611,233	14,450,067	Stationary	Unknown	1	26
10	2019/459 – IS	41,607,046	14,43,259	Stationary	Unknown	18	18
11	2019/469 – IS	41,628,083	14,43,705	Stationary	Unknown	18	18
12	2019/470 – IS	41,6,283	14,436,617	Stationary	Unknown	65	65
13	2019/471 – IS	41,626,483	14,449,983	Stationary	Unknown	3	13
14	2020/5 – IS	41,6,283	14,4,527	Semi-wild	**PASTURE**	1	12
15	2020/10 – IS	41,610,533	14,4,571	Extensive	**PASTURE**	41	41
16	2020/17 – IS	41,669,217	14,4,226	Semi-wild	**PASTURE**	3	69
17	2020/24 – IS	41,605,317	14,435,137	Stationary	**PASTURE**	34	34
18	2020/29 – IS	41,557,567	14,42,655	Stationary	**PASTURE**	3	160
19	2020/32 – IS	41,662,927	14,394,384	Extensive	**PASTURE**	8	8
20	2020/83 – IS	41,600,938	14,450,454	Semi-wild	**PASTURE**	14	14
21	2020/92 – IS	41,605,933	14,435,343	Semi-wild	**PASTURE**	1	69
22	2020/93 – IS	41,60,631	14,435,143	Semi-wild	**PASTURE**	1	53
23	2020/134 – IS	41,599,783	14,349,933	Extensive	**PASTURE**	1	27
24	2020/186 – IS	41,67,858	14,33,406	Stationary	Unknown	1	263
25	2020/281 – IS	41,613.55	14,4,488	Extensive	**PASTURE**	40	40
26	2020/354 – IS	41,6,127	14,452,283	Semi-wild	**PASTURE**	1	16
27	2020/391 – IS	41,609,426	14,325,026	Semi-wild	Unknown	1	20
28	2020/445 – IS	41,605,983	14,45,875	Semi-wild	**PASTURE**	1	29
29	2020/478 – IS	41,56,045	14,416,167	Semi-wild	**PASTURE**	1	34
30	2021/135 – IS	41,599,438	14,441,357	Transhumance	Unknown	58	58

**Figure 5:**
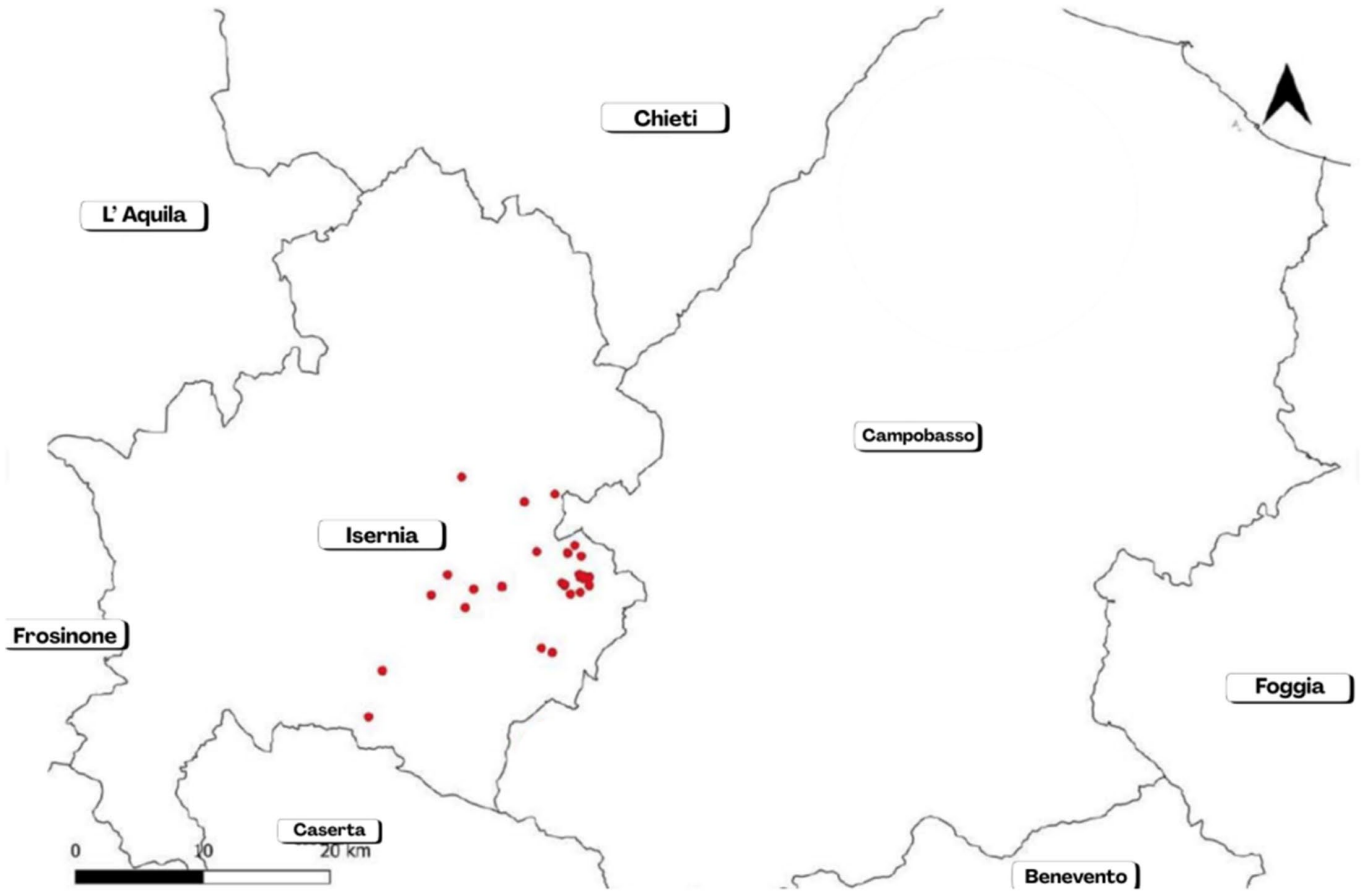
Map of outbreaks occurred in the province of Isernia during a re-emergence of cattle brucellosis in the years 2019–2021 (stopped since 2022).

Subsequent 16 outbreaks arose in 2020 (involving the province of Campobasso, the other province of Molise, through the sale of animals), during which 5 stamping-out operations occurred. In the following years, only one outbreak arose in 2021, on 12,962 animals tested and representing the 99.77% of 12,992 animals included in the eradication programme and hosted in 432 establishments. Outbreaks are overall reported in Table 3 and Figure 5. The brucellosis re-emergence stopped in 2022. In that year, 100% of the 12,792 bovine heads included in the eradication programme, and hosted in 406 establishments, was tested. Similarly, in 2023, 100% of the bovine heads included in the eradication was tested. The complete implementation (100%) of the serological control operations required by current regulations is a significant achievement, essential for promptly counteracting potential future resurgence of brucellosis in the province of Isernia.

In the province of Isernia, brucellosis affected 30 cattle herds; 15 outbreaks (50%) were registered as originating at grazing. The causative agent was identified as *B. abortus* biovar 3 in all cases. A total of 728 cattle heads were promptly culled.

In the Molise Region, no data concerning *Brucella* spp. in wildlife are available.

The province of Isernia is home to 718 water buffalo heads located in the PDO Mozzarella di Bufala Campana area (data registered in 2024). Buffalo establishments remained unaffected, saving this small yet significant livestock heritage in the province of Isernia since 1960.

**Figure 6:**
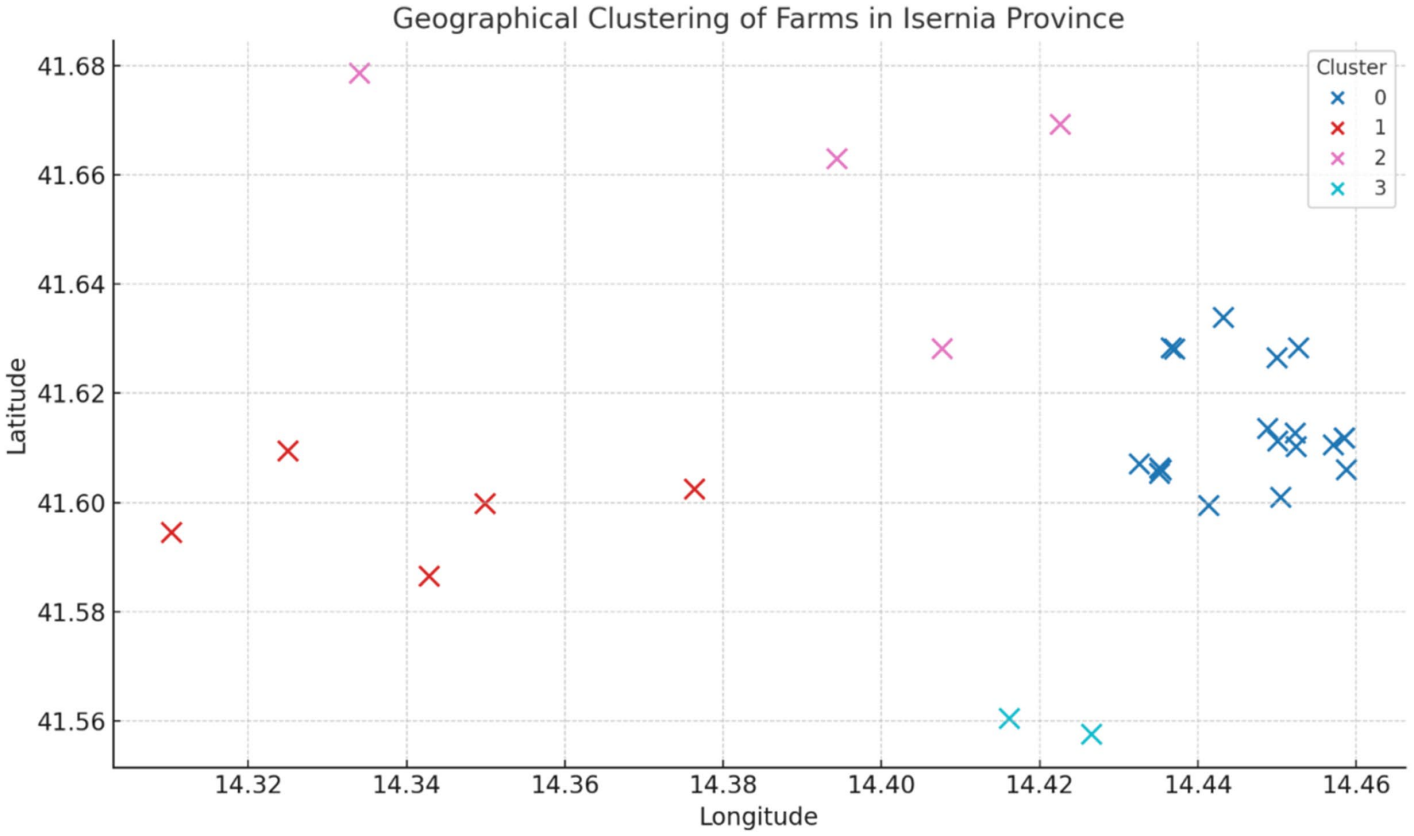
Geographical clustering of infected farms in the province of Isernia in the period 2019–2023.

The cluster analysis identified four distinct geographical clusters among the farms located in the province of Isernia (Figure 6). This spatial aggregation suggests that outbreaks tend to occur in geographically close groups.

### 3.3 Results concerning the provinces of Caserta and Salerno

The provinces of Caserta and Salerno boast a long and prestigious tradition in water buffalo *(Bubalus bubalis)* farming, a high-level zootechnical heritage.

The province of Caserta hosts the highest number of water buffaloes and buffalo establishments in Italy, despite a declining trend: between 2019 and 2023, the number of buffaloes decreased from 207,928 to 176,423 heads, while buffalo establishments decreased from 745 to 713 (Table 4).

**Table 4:** Number of water buffaloes and buffalo establishments in the province of Caserta in the period 2019–2023 (VETINFO).

Year	Number of water buffaloes	Number of buffalo establishments
2019	207,928	745
2020	205,695	724
2021	189,364	740
2022	175,185	721
2023	176,423	713

The province of Salerno hosts a slightly lower number of both buffaloes and buffalo establishments than Caserta, with a fluctuating trend in the period 2019–2023 (Table 5).

**Table 5:** Number of farmed buffaloes and buffalo establishments in the province of Salerno in the period 2019–2023 (VETINFO).

Year	Number of water buffaloes	Number of buffalo establishments
2019	103,952	352
2020	108,273	355
2021	112,690	388
2022	101,948	398
2023	104,851	369

In 21 municipalities of the province of Caserta, buffalo density exceeds 100 heads/km^2^, and in 9 municipalities farms with more than 1,000 buffaloes are present, with herd sizes ranging from 1,001 to 3,573 heads. The highest buffalo density is recorded in the municipality of Santa Maria La Fossa, that hosts 11,785 buffaloes within a municipal area of 25.35 km^2^ presenting a density of 3,682 heads/km^2^.

**Figure 7:**
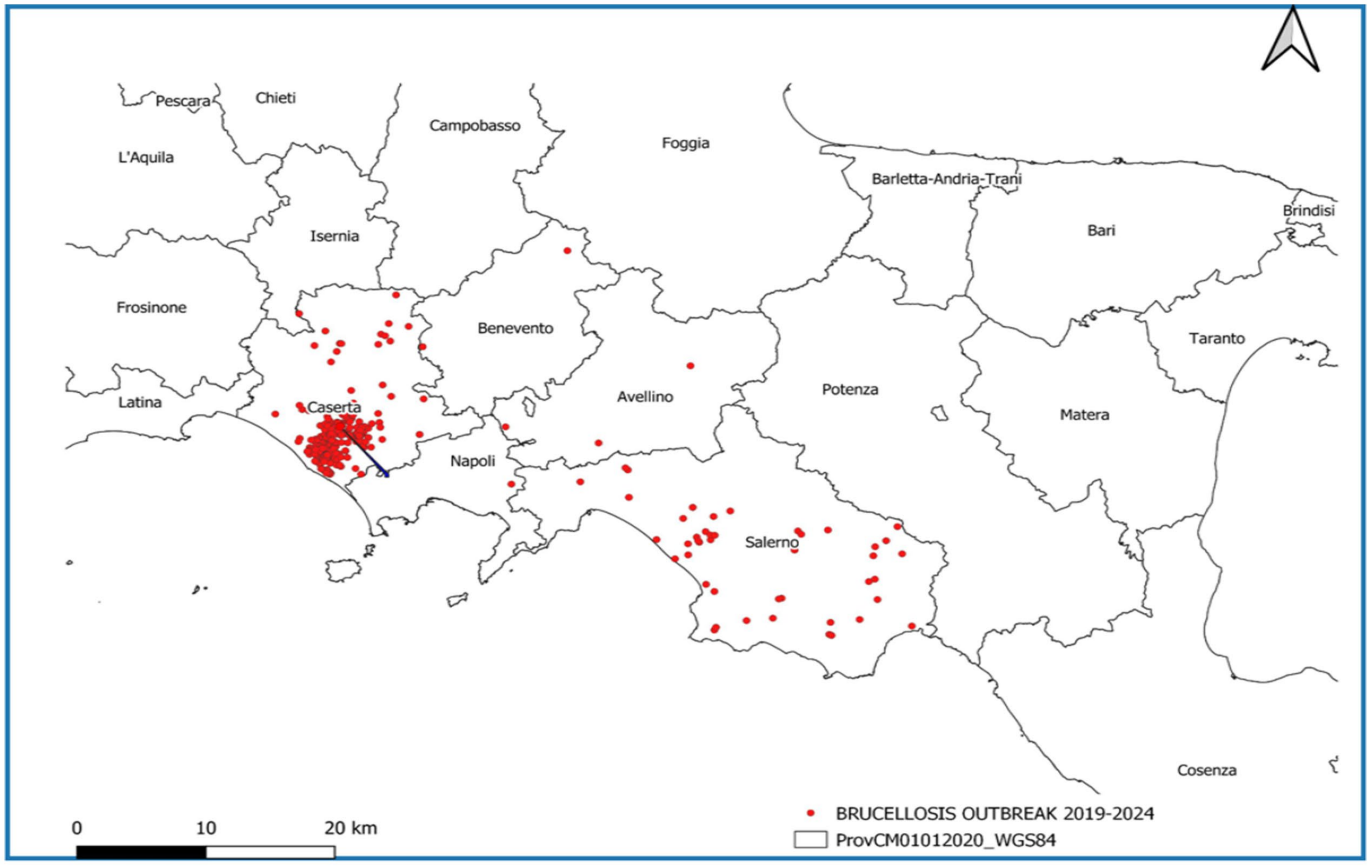
Map of buffalo brucellosis outbreaks in the Campania Region in the period 2019–2024.

**Figure 8:**
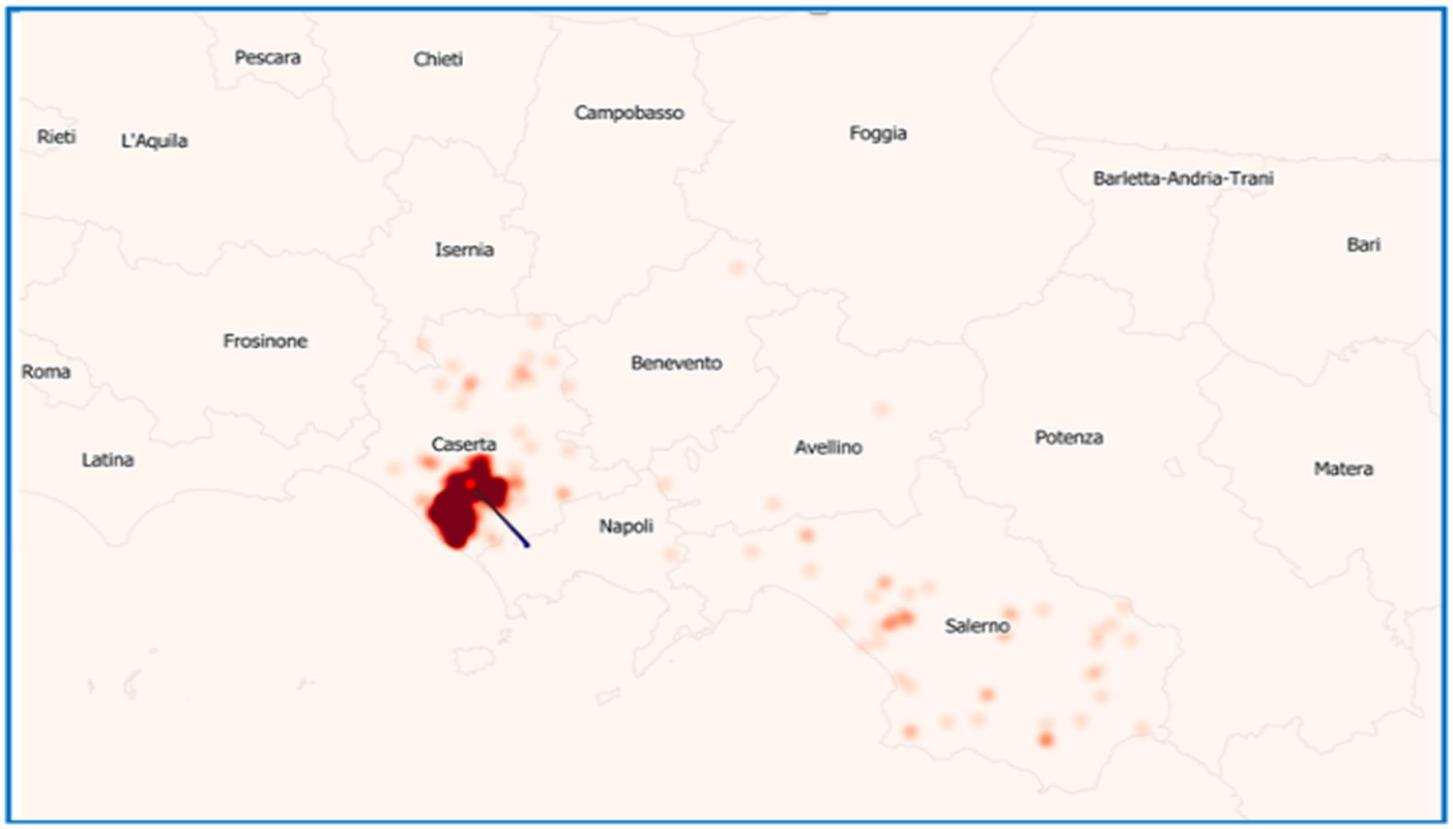
Map of density registered in buffalo brucellosis outbreaks in the Campania Region in which more intense colored areas correspond to a higher density of establishments where outbreaks occurred.

In the considered period, the province of Caserta recorded the highest number of outbreaks of brucellosis in Italy, followed by the province of Salerno (Figure 7). Outbreaks were primarily concentrated in the municipalities of Santa Maria La Fossa, Cancello ed. Arnone, Castel Volturno, and Grazzanise, once marshy areas and nowadays hosting the higher buffalo population. They are classified as infection clusters since buffalo brucellosis outbreaks have been repeatedly concentrated over the years with great intensity (Figure 8) and are subject to a dedicated eradication programme (Supplementary files S4 – Campania Region Rules for Cluster Areas, S5 – Order No. 1/2025 of the National Extraordinary Commissioner & Campania Region DG 104/2022).

In the other provinces of Campania, namely Naples, Avellino, and Benevento, only a few outbreaks were observed (Figure 7).

In the period 2019–2023, the province of Caserta recorded the highest number of positive heads in Italy, ranging from 8,766 positive heads in 2019 to 6,164 positive heads in 2023, with a peak of 11,930 positive heads in 2020. Positive results from serological testing were confirmed through isolation of the infectious agent from milk, vaginal swabs, or organ samples. The calculated prevalence in the buffalo population ranged from 4.78% in 2019 to 3.5% in 2023, with a peak of 6.63% in the year 2020 (Table 6). Details on prevalence, incidence, and stamping out concerning outbreaks of buffalo brucellosis that occurred in the province of Caserta in the period 2019–2023 are shown in Table 7.

**Table 6:** Number of positive heads in the province of Caserta and prevalence of brucellosis in the buffalo population in the period 2019–2023 (SIMAN).

Year	Number of positive heads	Prevalence in the buffalo population (%)
2019	8,766	4.78
2020	11,930	6.63
2021	8,943	5.04
2022	9,393	5.40
2023	6,164	3.50

**Table 7:** Details concerning outbreaks occurred in the province of Caserta in the period 2019–2023.

Year	Prevalent outbreaks	Incident outbreaks	Prevalence (%)	Incidence (%)	Stamping out
2019	85	63	11.47	8.50	17
2020	106	75	14.64	10.36	14
2021	131	87	17.70	11.76	30
2022	92	53	12.76	7.35	12
2023	81	51	11.36	7.15	10

In this province, a mandatory vaccination programme with the RB51 strain, a rough (R) *Brucella abortus* stable vaccine strain (Cloeckaert et al., 2002), is specific to the infection cluster municipalities (Campania Region, 2022).

**Figure 9:**
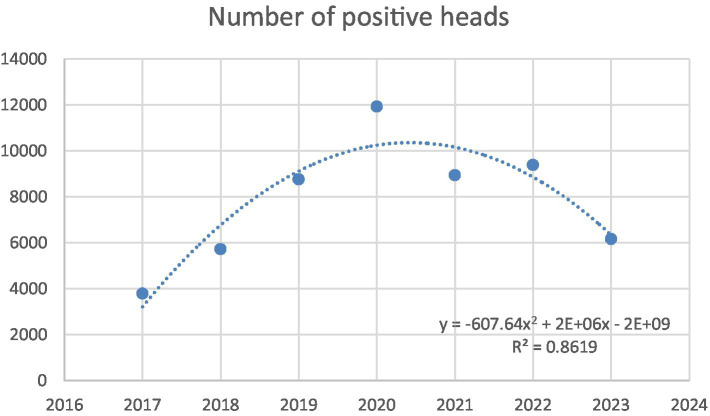
Quadratic regression model fitted to the number of positive heads in Caserta in the period 2017 to 2023.

The quadratic regression analysis was conducted to model the trend in the number of positive heads registered in Caserta over the period 2017–2023 (Figure 9). Data relating to the years 2017–2018 (Mazzeo et al., 2023) have been incorporated to improve the reliability of the regression model. The curve illustrates the temporal trend, highlighting an initial increase followed by a marked decrease in recent years. The model demonstrates a strong goodness of fit (*R*^2^ = 0.86) and statistical significance (*p* < 0.05) indicating a reliable trend over time.

During the period under consideration, the province of Salerno recorded a significantly lower number of buffalo brucellosis outbreaks and related positive animals compared to Caserta. The disease trend shows a decline, with no outbreaks reported in 2022 and 2023 (Table 8). Details on prevalence, incidence, and stamping out related to outbreaks in the province of Salerno from 2019 to 2023 are provided in Table 9.

**Table 8:** Number of positive buffalo heads in the province of Salerno and prevalence in the buffalo population in the period 2019–2023 (SIMAN).

Year	Number of positive heads	Prevalence in the buffalo population (%)
2019	369	0.35
2020	16	0.01
2021	36	0.03
2022	0	0.00
2023	0	0.00

**Table 9:** Details concerning outbreaks occurred in the province of Salerno in the period 2019–2023.

Year	Prevalent outbreaks	Incident outbreaks	Prevalence (%)	Incidence (%)	Stamping out
2019	11	9	3.12	2.56	1
2020	3	2	0.85	0.56	0
2021	6	5	1.55	1.29	0
2022	0	0	0.00	0.00	0
2023	0	0	0.00	0.00	0

Positive cases from Caserta and Salerno provinces were analyzed using SVTT analysis, that reveals a marked divergence in the temporal patterns of positive heads between the provinces of Salerno and Caserta over the 2019–2023 period. In Salerno, the data demonstrate a sharp and consistent decline, with positive cases dropping from 369 in 2019 to zero by 2022 and 2023. In contrast, Caserta exhibits a much higher prevalence, peaking at nearly 12,000 cases in 2020, followed by moderate fluctuations and a reduction to approximately 6,000 in 2023. Despite the downward trend, the persistence of a relatively high number of cases indicates continued challenges in disease management.

In outbreaks occurring over the years in the province of Caserta, *Brucella abortus* biovar 1 (significantly associated with water buffalo) and biovar 3 (significantly associated with cattle) have consistently been identified in water buffalo. However, 99.9% of the outbreaks were caused by *B. abortus* biovar 1. Orrù et al. (2024) found that the low genetic diversity of *B. abortus* in the Campania Region was further confirmed through Multilocus Sequence Typing (MLST) analysis. The cluster analysis they performed revealed that both the strains sequenced in their study and those isolated in Campania in 2022 grouped into three clusters that have been circulating since 2017. These three *B. abortus* clusters were responsible for the recurrent outbreaks affecting livestock establishments in the province of Caserta.

In the Campania region, a wildlife surveillance monitoring plan is in place. To date, no positive cases have ever been detected, with the sole exception of one case in January 2025 involving a wild boar in the province of Benevento (data no shown).

## 4 Discussion

Despite their proximity, the studied areas differ not only in their geographical and environmental characteristics but also in their cultural heritage and farming traditions. These differences influence the livestock systems in each territory.

The province of Isernia has a low density of establishments, mostly in the mountains, and family-run farming. Furthermore, seasonal movement of animals is still practiced, with livestock moving from lowland and hilly pastures to mountain grazing areas during the summer. In Molise, in 2024, 328 pastures were registered, with the greatest number occurring in the province of Isernia (VETINFO). Cattle farming is crucial for the production of PDO pasta filata cheeses, such as “Fior di Latte Molisano” (GURI, 2024) and “Caciocavallo Silano” (Commission Regulation, 1996). These artisanally produced cheeses contribute significantly to the local economy, attracting tourists looking for experiences linked to ancient traditions (Moretti et al., 2023).

In Molise, cattle brucellosis outbreaks had a clear and constant decline in recent years. In 2022 and 2023, no outbreaks occurred in the province of Isernia. By now, only the province of Campobasso maintains the status of bovine brucellosis DFS. Isernia is in the process of acquiring DFS according to the Commission Delegated Regulation (EU) (2020), Annex IV, establishing that 99.8% of brucellosis-free establishments and 99.9% of brucellosis-free animals have tested negative, and that no confirmed cases of infection with *Brucella abortus*, *B. melitensis*, or *B. suis* in cattle have been recorded in the previous three years. A derogation concerns a single outbreak that occurred and was conclusively resolved within the calendar year in which it arose.

Regarding the brucellosis reemerged in the province of Isernia, 50% of cases originated in the pasture, potentially resulting through interaction with other infected animals coming from neighboring non-DFS provinces. The spatial aggregation obtained with cluster analysis, suggesting that outbreaks occur in geographically close groups, highlights the importance of localized surveillance and containment strategies. The clusters probably reflect shared grazing areas, common routes of animal movement, or similar management practices, all of which may influence the transmission dynamics of brucellosis. In many areas of southern Italy, the tradition of transhumance, which seasonally moves livestock from the plains to the mountains and vice versa, has been largely abandoned due to changes in both farming systems and the lifestyle of farmers. But the practice of vertical transhumance, in which livestock move to close mountain pastures during the summer, persists, helping to reduce feed costs and improve milk quality thanks to the mountain flora. Despite the beneficial effects, nomadic and mixed-use grazing areas are more susceptible to the spread of *Brucella* spp. especially due to the interaction between domestic and infected wild animals (Lokamar et al., 2022). Furthermore, animals coming from farms in non-DFS areas, which may have acquired the infection recently, resulting seronegative in diagnostic tests, may become excretory during the grazing period, with subsequent transmission of the infection.

Regulation (EU) 2016/429, in force since April 21, 2021, which is being transposed into Italian law through a series of implementing decrees, mandates the control of brucellosis in wildlife. As a result, sufficient data to accurately assess the impact of wildlife on the transmission of brucellosis to farmed animals at the national level will only be available in a few years. For now, the available data stem from non-uniform but relevant studies (De Massis et al., 2019; Parolini et al., 2024; Ruano et al., 2025).

The Ministerial Decree of May 2, 2024, currently in force, pays particular attention to the containment of brucellosis among livestock authorized for grazing (Supplementary file S2). This is undoubtedly irrelevant in those infection cluster municipalities of the Province of Caserta where grazing is prohibited in most areas due to the high prevalence and incidence of brucellosis; however, it is of fundamental importance for farms in the Province of Isernia, which have traditionally benefited from access to pasture. In the future, it will be possible to obtain sufficient data to assess the effectiveness of the measures enacted in 2024.

The Provinces of Caserta and Salerno are leaders in the production of PDO Mozzarella di Bufala Campana, a very famous fresh pasta filata cheese made from water buffalo milk. Here, buffalo farming and mozzarella production are not only economic pillars but also integral elements of local identity, gastronomic and cultural traditions (Levante et al., 2023).

Therefore, the agro-economy is deeply influenced by these typical and traditional dairy products. Since brucellosis can be transmitted to humans through the consumption of dairy products, outbreaks of cattle and buffalo brucellosis could have a significant impact not only on animal and human health, put at risk through direct and indirect contacts with infected animals and the contaminated environment, but also on food safety and the local economy. For these reasons, it is crucial not only to monitor the number and extent of brucellosis outbreaks in farms across these territories but also to track their evolution over time.

This approach helps assess whether the measures implemented by farmers, policymakers, veterinary health institutions, and local health authorities are effective in controlling the disease. In the provinces of Caserta, before grazing prohibition in cluster and buffer zones, only 2 outbreaks originated from grazing in 2019. It can therefore be inferred that grazing is not a determining factor in the transmission of *Brucella* spp. in the province of Caserta. Instead, flooding events appear to cause widespread environmental contamination, introducing the pathogen into farms even when animals do not cross farm boundaries, have no direct contact with animals from other farms, and do not share spaces that could facilitate indirect transmission. Flood mitigation measures implemented in the province of Caserta (Supplementary files S4, S5) appear to be contributing effectively to disease control, as evidenced by a continuing, though fluctuating, decline in buffalo brucellosis outbreaks and affected animals. This decreasing trend (Figure 9) may reflect the positive impact of regional eradication efforts. These initiatives, coordinated under the Campania Region’s mandatory programme (Campania Region, 2022) and with the expected insights by Order No. 1/2025 from the National Extraordinary Commissioner on Strengthening Measures for Eradicating Water Buffalo Brucellosis in the Province of Caserta (Italian Ministry of Health, 2025), whose results will be available in the future, highlight the strategic management of drainage canals as a key element in controlling buffalo brucellosis transmission. The province of Salerno has brought brucellosis cases down to zero, as highlighted by the data in Table 8, which shows a complete absence of disease since 2022. This trend suggests the successful implementation of effective control measures, including enhanced biosecurity protocols and adjustments to surveillance and testing strategies. In contrast, the province of Caserta continues to report persistent, though gradually declining, cases. This spatial heterogeneity in temporal trends can only be partially explained by environmental and geographical factors.

Climatic conditions and topographical features are known to significantly influence the survival, transmission, and dissemination of infectious agents. Notably, Caserta is characterized by substantially higher livestock density, both in terms of the number of farms and the number of animals per farm, compared to Salerno. This higher concentration putatively increases transmission pressure and facilitates the spread of infection. The SVTT analysis underscores the importance of territory-specific surveillance and intervention strategies. A comprehensive understanding of local ecological, geographical, and production system characteristics is essential to inform tailored disease control programmes and ensure efficient resource allocation. An example of large-scale implementation of biosecurity and measures aimed at infection prevention is provided by the Classyfarm System operating in Italy through the VETINFO portal. Classyfarm is the official surveillance platform developed by the Ministry of Health to monitor livestock farms and classify them based on their level of risk. It focuses primarily on key areas such as animal welfare, biosecurity measures adopted in the establishments, slaughterhouse outcomes, and the use of antimicrobials and data concerning antimicrobial resistance. The system is designed to handle and integrate large volumes of data from multiple sources, leveraging business intelligence tools to perform comprehensive analysis and support evidence-based decision-making.

Despite our primary focus is on buffalo farming in Campania and cattle farming in the province of Isernia, in the province of Salerno a high rate of brucellosis transmission among grazing cattle was observed, emerging only as a secondary result of this study, but it represents a concern. Three of the five provinces of the Campania Region, Benevento, Salerno, and Naples, acquired the DFS for ovine and caprine brucellosis. The provinces of Avellino and Caserta have met the requirements set by Commission Delegated Regulation (EU) (2020) to apply for DFS at the provincial level. Sporadically, outbreaks caused by *B. melitensis* occurred in ovine and caprine species (Avellino, 2021; Benevento, 2021; Naples, 2020) and were promptly extinguished. In the province of Salerno, although officially free, a brucellosis outbreak arose in 2024. It originated from the movement of animals from the Puglia Region and involved human cases.

The reported findings reinforce the fundamental principle of the One Health approach (FAO, UNEP, WHO, and WOAH, 2022), which emphasizes the importance of targeted, context-specific interventions developed through collaboration among institutions, cross-sectoral professionals, and, in the case of buffalo farming, the farmers themselves. Within this framework, fostering mental health by mitigating economic uncertainty and stress emerges as a critical component (Mazzeo et al., 2022). This critical multidisciplinary approach, established under the One Health framework, was formally endorsed by national legislation in 2024, following its earlier adoption by the Campania Region in 2022. Over time, significant results have been achieved, particularly in the province of Salerno, which had already made substantial progress toward the eradication of *Brucella melitensis* infection in small ruminants. More recently, notable advances have also been recorded in the province of Caserta, primarily due to targeted vaccination campaigns implemented in municipalities with a historical presence of the disease and characterized by a higher buffalo density per square kilometer. These achievements have been further reinforced by improved manure management practices and increasingly precise maintenance of the regional hydrographic network.

Although no foodborne human case occurred in the EU in 2023 (EFSA and ECDC, 2024), sporadic outbreaks arise among consumers of unpasteurized dairy products, especially cheese (Islam et al., 2023), which are often also those with protected designations. To save their prestigious quality, DFS of the production zone would be of great importance.

In this context, the adoption of biosecurity measures in pastures, such as fences, nets, or other structures to divide grazing areas by species, origin, and health status, would limit contact with wildlife and the promiscuity of animals from different farms. These prevention and control measures would mitigate the risk of contagion without limiting the intake of the precious herb of the Apennine flora and the cost savings related to feed and water.

The adoption of advanced technologies to monitor the movements and interactions of animals (Sutherland et al., 2018; Tobin et al., 2020; Odintsov Vaintrub et al., 2021; Lv and Li, 2022; Džermeikaitė et al., 2023; Zhang et al., 2023; Hlimi et al., 2024; Mohite et al., 2024; Manville et al., 2024) and early detection of pathogens and health issues (Jaeger et al., 2019; Chang et al., 2022) could also help control brucellosis mitigating impacts on animal populations and ecosystems in case of high establishment density (McCorkell et al., 2014; Aquilani et al., 2022; Alipio and Villena, 2023). The “Campania-GPS,” a Campania Regional Project, has been launched in order to experiment, among other initiatives, a sentinel monitoring system estimating the representative average movement patterns of entire cattle herds by utilizing a limited number of GPS devices embedded in ear tags. Initial results have demonstrated that applying these devices to lead animals, in authorized grazing areas, enables detailed tracking of the entire herd’s movements. The findings reveal intermingling among animals from different farms and indicate that livestock may occasionally stray onto land not owned by their respective farms.

The assiduous serological self-monitoring of the herd carried out through Milk-ELISA on pooled samples (Supplementary file S3), enabling the prompt detection of the presence of infected animals, is of the utmost importance. It can limit the potential risk due to undetected brucellosis spreading within herds during the intervals of one or more years between official controls.

Several recommendations emerged from recent studies addressing various aspects of BioDistrict, described as a geographically defined area where farmers, businesses, local authorities, and communities collaborate to promote sustainable agricultural practices based on organic farming principles with the goal of integrating organic production with environmental conservation, local food systems, rural development, and eco-friendly tourism, fostering a circular economy that benefits both people, and the environment (Belliggiano et al., 2024; Mazzeo et al., 2024; Sturla et al., 2024).

The approach necessitates the active involvement of key stakeholders, including researchers, public health authorities, and policymakers, and must be supported by substantial financial investments. Critical components include the strengthening of biosecurity measures, the implementation of advanced animal traceability systems, continuous professional training programmes (also extended to farmers), expert-driven information-sharing technologies, and the promotion of voluntary serological self-monitoring practices. The use of modern technologies to disseminate information and operational guidelines accessible to all would greatly enhance farmer engagement (Paixão et al., 2024), especially if the economic value or return of well-executed actions can be demonstrated (Auplish et al., 2024).

However, these initiatives are costly and the burden of implementing them cannot fall solely on farmers, especially in struggling inner areas facing decline, depopulation, and infrastructural, service, and technological gaps (Mazzeo et al., 2024). Consequently, the Quadripartite Organization – FAO, UNEP, WHO, and WOAH – in the One Health Joint Plan for Action 2022–2026, underlines that the following key actions should be prioritized: enhance financial support by securing sustainable and efficient funding mechanisms at global, regional, and national levels; strengthen stakeholder engagement to ensure inclusive, coordinated actions; foster strategic collaborations by identifying opportunities for deeper partnerships and synergies across sectors; adopt a unified resource mobilization strategy, developing a structured, coordinated approach that leverages both public and private sector contributions (FAO, UNEP, WHO, and WOAH, 2022).

## Data Availability

The original contributions presented in the study are included in the article/Supplementary material, further inquiries can be directed to the corresponding authors.

## Footnotes

^1^https://www.izs.it/IZS/Engine/RAServePG.php/P/256210010520/L/1 (Accessed April 27, 2025).

^2^https://www.vetinfo.it/ (Accessed March 21, 2025).

^3^https://www.vetinfo.it/j6_statistiche/#/ (Accessed April 29, 2025).

^4^https://wahis.woah.org/#/dashboards/control-measure-dashboard (Accessed March 21, 2025).
