# Supplementary material for: Brucellosis in cattle and buffalo in southern Italian provinces: trends in presence of territory-specific One Health measures
Source: Front Microbiol. 2025 Jun 6;16:1609336. doi: 10.3389/fmicb.2025.1609336 (PMC12179989; doi:10.3389/fmicb.2025.1609336)
Supplement: Supplementary file 1 [file Data_Sheet_1.pdf]

DATA SHEET S1

TRANSPLACENTAL AND BREAST-FEEDING VERTICAL TRANSMISSION IN CATTLE

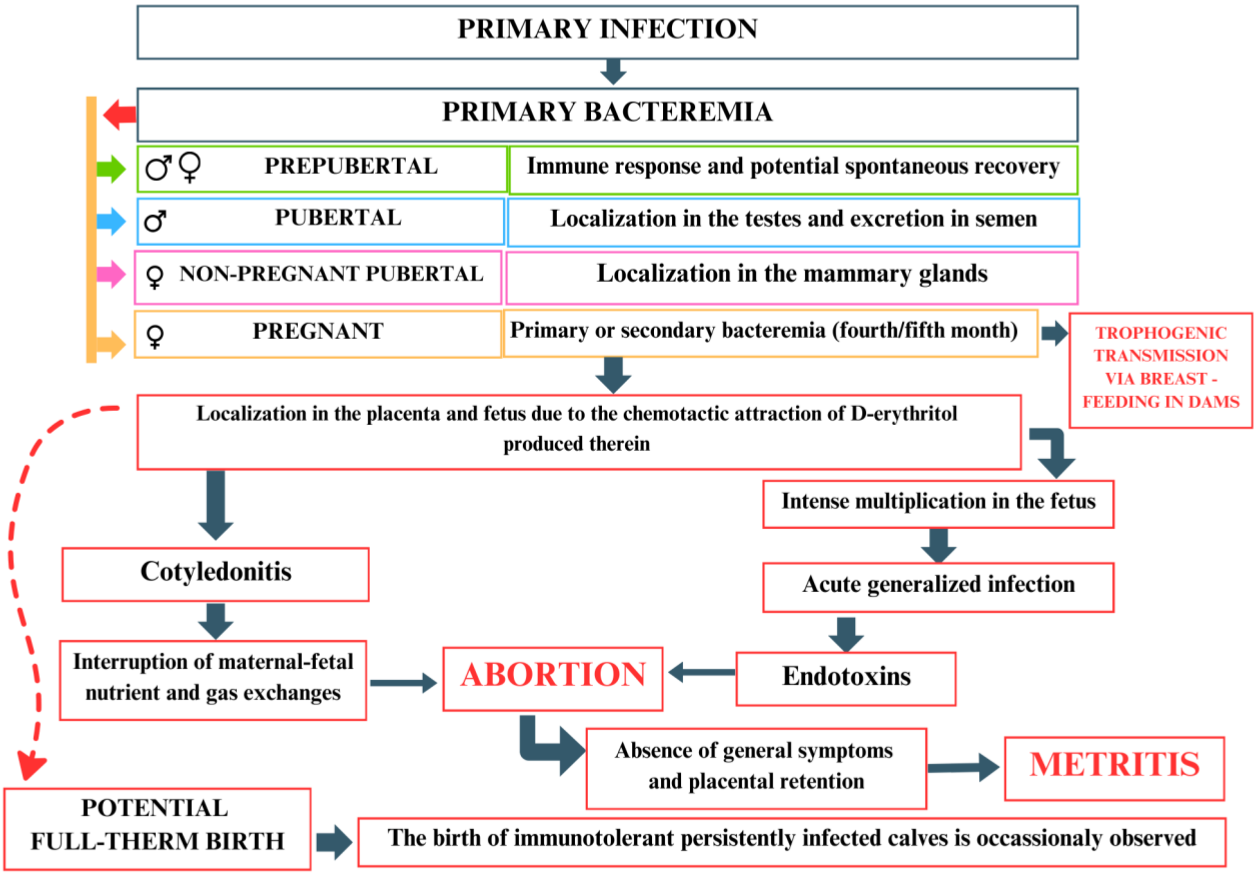

Reference

Mazzeo A. (2010). Il Controllo delle Principali Zoonosi e Malattie Infettive in Produzione Primaria-Elementi di Infettivologia ed Esempi di Profilassi, Aracne Editrice, Roma (IT)

Graphic editing: Sebastiano Rosati
