# Supplementary material for: Brucellosis in cattle and buffalo in southern Italian provinces: trends in presence of territory-specific One Health measures
Source: Front Microbiol. 2025 Jun 6;16:1609336. doi: 10.3389/fmicb.2025.1609336 (PMC12179989; doi:10.3389/fmicb.2025.1609336)
Supplement: Supplementary file 2 [file Data_Sheet_2.pdf]

S2

EU and Italian Regulatory Frameworks on Animal Health and Grazing Practices

Regulatory framework in the EU

The Regulation (EU) 2016/429 introduced comprehensive rules for managing and preventing transmissible animal diseases, including zoonoses.

Commission Implementing Regulation (EU) 2018/1882 categorizes the listed animal diseases - for the listed animal species - in five categories, ranging from A up to E (CIR, 2018).

Specifically, *Brucella abortus, B. melitensis*, and *B. suis* infections are categorized as:

- B+D+E disease categories in *Bison* spp., *Bos* spp., *Bubalus* spp., *Ovis* spp., and *Capra* spp. In particular, B diseases are those that must be controlled with the goal of eradicating them throughout the EU. In these species, infections are classified also as D disease category - for which measures are needed to prevent the spreading on account of its entry into the EU or movements between MSs - and E disease category, that includes listed diseases for which there is a need for surveillance within the EU.
- D+E disease categories in other species of the Order of *Artiodactyla*.
- E disease category in *Perissodactyla*, *Carnivora* and *Lagomorpha*.

Commission Delegated Regulation (EU) 2020/689 defines the conditions necessary to grant, maintain or suspend the DFS achieved through eradication or surveillance mandatory programmes in bovine animals - ungulates belonging to the genera *Bison*, *Bos* (including the subgenera *Bos, Bibos, Novibos, Poephagus*) and *Bubalus* (including the subgenus *Anoa*) and the offspring of crossing of those species – in ovine animals intended as ungulates belonging to the genus *Ovis,* and caprine animals intended as ungulates belonging to the genus *Capra,* and the offspring of crossing of both those species. Annual mandatory surveillance programmes are carried out in DFS zones to confirm the disease-free status; non-DF zones must implement mandatory programmes for brucellosis eradication. The competent authority may include, in eradication programmes, vaccination of listed species, vaccination of an additional farmed animal population, and vaccination of wild animals.

Commission Implementing Regulation (EU) 2020/2002 defines notification and reporting requirements and delegates the European Commission to approve disease DFS for MSs or their specific areas. These measures aim to tackle brucellosis spreading in farmed animals and wildlife, and to reduce the risk to public health (CIR, 2020).

Regulatory framework concerning grazing practices in Italy

The Decree of 2 May 2024 of the Italian Minister of Health implements mandatory national eradication programmes for brucellosis in bovine, ovine, and caprine animals. Its Annex 1 identifies two primary threats to the eradication of brucellosis: the mixed-use grazing – that involves georeferenced grazing areas where animals from multiple establishments are mixed without separation, creating a single, high-risk epidemiological unit – and the nomadic grazing, concerning a practice of rearing cattle, sheep, and goats in a migratory manner, utilizing public or private land across one or more municipalities.

The Decree establishes guidelines for the movement of animals from non-DF provinces to DF Provinces, in order to preserve their previously acquired DF status. It is mandatory that:

- Any bovine older than 12 months of age must test negative to a serological test performed within 30 days before the movement.

- All animals of diagnostic age test negative to the Rose Bengal Test (RBT) and Complement Fixation Test (CFT) carried out in the establishment within the last six months.
- Infection prevalence in the province where the establishment is located is below 2%.

The rules for moving animals, particularly for activities such as transhumance and extensive livestock farming, are very strict and aimed at preventing the spread of disease and requires specific veterinary service responsible for the destination establishment to recommend or deny animal entry into the jurisdiction destination.

Transhumance, mountain pasturing, and alpine pasturing refer to the seasonal movement of animals from plains to mountainous regions and *vice versa*. Extensive and semi-extensive farming involves rearing animals on farmland located in provinces other than the one of residence. All those movements are authorized only for pastures that are geo-referenced and registered in the National Data Bank database (VETINFO).

The shared use of pastures by animals from both non-free and free provinces is prohibited.

Furthermore:

- Operators of an establishment in a DFS province wishing to move animals to a pasture in a non-DF province must ensure the fencing/boundary of the destination pasture.
- Pastures located in infection clusters must always be fenced/bounded.
- Animals from multiple establishments are treated as a single high-risk epidemiological unit.

Within 30 days of the animal's return from pasture, local veterinary services must test them while keeping them apart from the rest of the herd; if not, the entire herd must be tested.

Interregional movements for transhumance, grazing, mountain pasturing, and alpine pasturing are prohibited in areas classified as infection clusters, defined by territorial contiguity with confirmed cases and presumed origin from a common source.

To maintain DF status:

- 99.8% of establishments must remain disease-free.
- 99.9% of animals must remain disease-free.
- In the last 12 months, no confirmed cases of infection with *Brucella abortus*, *Brucella melitensis*, or *Brucella suis* must have been recorded in bovines or sheep/goats.

In non-DF territories, animals involved in transhumance, pasturing, mountain pasturing, or raised extensively/semi-extensively - unless already electronically identified - must be marked with an endoruminal bolus or other identification method linked to genetic material collection, at the operator's expense. The costs of testing and analyses are sustained by the operator, except in cases where they coincide with the tests included in the national eradication programme. All activities must be managed by the Veterinary Officers through the VETINFO portal.

Key EU and Italian Laws on Animal Health and Grazing Practices in Chronological Order

Regulation (EU) 2016/429 of the European Parliament and of the Council of 9 March 2016 on transmissible animal diseases and amending and repealing certain acts in the area of animal health (‘Animal Health Law’). Official Journal of the European Union, L 84, 31 March 2016. https://eur-lex.europa.eu/eli/reg/2016/429/oj

(CIR) Commission Implementing Regulation (EU) 2018/1882 of 3 December 2018 on the application of certain disease prevention and control rules to categories of listed diseases and establishing a list of species and groups of species posing a considerable risk for the spread of those listed diseases. *Official Journal of the European Union*, L 308, 4 December 2018. https://eur-lex.europa.eu/legal-content/EN/TXT/PDF/?uri=CELEX:32018R1882

(CIR) Commission Implementing Regulation (EU) 2020/2002 of 7 December 2020 laying down rules for the application of Regulation (EU) 2016/429 of the European Parliament and of the Council with regard to Union notification and Union reporting of listed diseases, to formats and procedures for submission and reporting of Union surveillance programmes and of eradication programmes and for application for recognition of disease-free status, and to the computerised information system. Official Journal of the European Union, L412, 8.12.2020. https://eur-lex.europa.eu/legal-content/EN/TXT/PDF/?uri=CELEX:32020R2002

Commission Delegated Regulation (EU) 2020/689 of 17 December 2019 supplementing Regulation (EU) 2016/429 of the European Parliament and of the Council as regards rules for surveillance, eradication programmes and disease-free status for certain listed and emerging diseases. Official Journal of the European Union, L 174, 3 June 2020. https://eur-lex.europa.eu/legal-content/EN/TXT/PDF/?uri=CELEX:32020R0689

Italian Ministry of Health. Decree of May 2, 2024 – Adoption of the mandatory national programmes for the eradication of brucellosis and tuberculosis in cattle and brucellosis in sheep and goats (24A03318). Official Gazette of the Italian Republic No. 151 of June 29, 2024, pp. 17-20. https://www.gazzettaufficiale.it/eli/gu/2024/06/29/151/sg/pdf; https://www.izsler.it/tbcentro/wp-content/uploads/sites/19/2024/12/interno_IZS_Brucellosi.pdf

(CIR) Commission Implementing Regulation (EU) 2024/1332 of 17 May 2024 amending certain Annexes to Implementing Regulation (EU) 2021/620 as regards the approval or withdrawal of the disease-free status of certain Member States or zones or compartments thereof as regards certain listed diseases and the approval of eradication programmes for certain listed diseases O J EU, Series L, 21.05.2024, 1-7. https://eur-lex.europa.eu/legal-content/EN/TXT/PDF/?uri=OJ:L_202401332
