# Supplementary material for: Brucellosis in cattle and buffalo in southern Italian provinces: trends in presence of territory-specific One Health measures
Source: Front Microbiol. 2025 Jun 6;16:1609336. doi: 10.3389/fmicb.2025.1609336 (PMC12179989; doi:10.3389/fmicb.2025.1609336)
Supplement: Supplementary file 3 [file Data_Sheet_3.pdf]

S3

Diagnostic Tests Adopted for Brucellosis in Italy under the Ministry of Health Decree of May 2, 2024 and Future Perspectives

Serological Tests

RBT - According to the Manual of Standards of the World Organisation for Animal Health (WOAH, 2024) and under the Ministry of Health Decree of May 2, 2024 (Italian Ministry of Health, 2024), the Rose Bengal Test (RBT) - a simple spot agglutination test using antigen stained with Rose Bengal and buffered to pH 3.65 ± 0.05 - is used in Italy as a screening test for all species subject to the eradication programme.
The buffered *Brucella* antigen with Rose Bengal is produced by the IZS AM, which is also the National Reference Centre for Brucellosis (NRC) and National Reference Laboratory (NRL) for Brucellosis, adopting the procedures of the EU Reference Laboratory (EU-RL) for *Brucella* and the WOAH Manual of Standards.

CFT - The Complement Fixation Test (CFT) is used as a confirmatory test and for titration of sera positive in the screening test.
The CFT test must be performed on all samples positive to RBT and on all animals in the herd in the case of suspected herds, even if they tested negative for RBT.
The CTF test is a quantitative method, it is considered positive if the examined serum shows a titer equal to or greater than 20 IUFC/mL (International Unit Fixing the Complement).
If an animal's serum tests negative for both RBC and CFT, the animal is considered negative and not suspected of infection.
If an animal's serum tests positive for CFT (≥20 IUFC/mL) and is negative or positive for SAR, the animal is considered suspected of infection.
In all cases of suspected infection or uncertain results, the health qualification of the herd is suspended until the suspicion or diagnostic uncertainty is resolved.
When herds have a suspended health qualification due to one or more suspected cases of infection, the veterinary service initiates an epidemiological investigation and:

- Prohibits the movement of brucellosis-susceptible species into or out of the herd, except for immediate slaughter at a designated abattoir.
- Orders the isolation of suspected cases within the herd. If isolation is not possible or cannot be maintained for the necessary period, the veterinary service may order the slaughter of suspected animals to protect public health.

CFT-RB51 - When unauthorized use of RB51 vaccine – adopted in Italy and usable under specific authorization - is suspected in brucellosis-free provinces or herds, a specific Complement Fixation Test for RB51 (CFT-RB51) may be required to detect anti-R antibodies, as RB51 is a live vaccine based on a *Brucella abortus* rough (R) strain, which does not elicit the production of antibodies against smooth (S) *Brucella* spp. antigens detectable by the standard diagnostic tests RBT and CFT (Ciuchini et al., 2005; Cloeckaert et al., 2002).
The test sensitivity in cattle and buffaloes is approximately or above 90% in the first four months post-vaccination, decreasing over time in animals vaccinated prepubertally even with a triple dose and booster (as in buffaloes).
Animals testing positive for CFT-RB51 should not generally be slaughtered or sold. These animals

must undergo monthly testing with CFT-RB51 until they yield two consecutive negative results at least one month apart. Once the previously positive animals test negative, all other animals in the herd must be tested and return negative results for CFT-RB51, alongside wild-type brucellosis testing, which is required to restore the herd's status. After these results, milk production for human consumption without pasteurization may resume.

ELISA – Indirect ELISA in individual serum samples can be used as a complementary test or as required for export/import.

Milk-ELISA – The test in pooled or bulk milk samples can be used as a screening tool in unvaccinated DFS cattle herds from farms where at least 30% of lactating cows are in milk production, located in DFS Provinces. Confirmatory tests, in cases of positivity, must be conducted on serum samples collected from individual animals that contributed to the bulk milk pool.

Milk-ELISA in self-monitoring procedure

In Italy, testing activity for bovine brucellosis is an exclusive prerogative of the territorial IZS. However, with the Italian Ministry of Health Decree of May 2, 2024, third parties can conduct self-monitoring activity through the serological Milk-ELISA test on pooled milk samples from farmed cattle and buffaloes - collected by the farm veterinarians - but only if serological controls ensure that, while enabling the prompt detection of the presence of *Brucella* spp. infection, tests do not allow identification of positive animals. This measure aims to address the potential risk of undetected brucellosis spreading within herds during the intervals of one or more years between official controls. In the case of positivity, official controls must be performed on individual samples.

The milk-ELISA test on bulk or pooled samples can be adopted as part of self-monitoring, and milk sampling must be performed by the establishment veterinarian.

Microbiological and Molecular Tests

The microbiological isolation of *Brucella abortus*, *B. melitensis*, and *B. suis* must be performed on tissues and/or matrices of animals that tested positive in serological tests to confirm suspected brucellosis cases or in the presence of clinical signs consistent with brucellosis. The cultural examination for the detection of *Brucella* spp. must be carried out in a BSL3 laboratory.

In live animals, the target matrices for cultural isolation are milk, vaginal discharges (vaginal and cervical swabs), semen, synovial fluid, and, in cases of abortion, the fetus, amniotic fluid, and fetal membranes.

In *post mortem* examinations, the target organs for microbiological isolation are:

- Reticulo-endothelial tissues (spleen, supra-mammary lymph nodes, iliac lymph nodes, retro-pharyngeal lymph nodes).
- Mammary glands.
- Testes.
- Pregnant or post-partum uterus.

The National Reference Laboratory for Brucellosis has developed PCR and RT-PCR methods.

The phenotypic typing tests to be conducted include phage lysis, agglutination with monospecific anti-A antigen, anti-M antigen, or anti-R antigen sera, differential growth on media with fuchsin and thionin, H_2S production, and CO_2 requirement, following the EURL-SOP "*Brucella* typing" (Revision 01, June 2021).

Whole Genome Sequencing (WGS) cannot fully replace the traditional phenotypic typing methods that determine the identification of *Brucella* species and biovars according to conventional

classification.

Future Perspectives

The evolution of regulatory frameworks may lead to advancements in screening test development, particularly the adaptation of indirect ELISA to meet emerging diagnostic needs by enhancing specificity, sensitivity, and practicality, as experimental devices tested in multiple serodiagnosis or in multiple antigen detection (Petracca et al., 2004; Ermolli et al., 2006; Ermolli et al., 2008). The performance of diagnostic tests is variable in different epidemiological settings (Baruch et al., 2020; Bodenham et al., 2021) and determining if pooled or bulk tank milk is the best approach for a disease management programme should be carefully considered (Brito and Hick, 2023). Furthermore, the indirect ELISA test in pooled sera samples may be an appropriate method for bovine brucellosis screening at slaughterhouse (Bodenham et al., 2020).

References

Baruch J., Suanes A., Piaggio J.M., Gil A. D. Analytic Sensitivity of an ELISA Test on Pooled Sera Samples for Detection of Bovine Brucellosis in Eradication Stages in Uruguay. *Front. Vet. Sci.* 2020, 7. https://www.frontiersin.org/journals/veterinary-science/articles/10.3389/fvets.2020.00178/full

Bodenham R. F., Mazeri S., Cleaveland S., Crump J. A., Fasina F. O., de Glanville W. A. et al, Latent class evaluation of the performance of serological tests for exposure to *Brucella* spp. in cattle, sheep, and goats in Tanzania. *PLoS Negl. Tropical Dis.*, August 2021. https://doi.org/10.1371/journal.pntd.0009630

Brito, B. and Hick, P., Milk as a diagnostic fluid to monitor viral diseases in dairy cattle. *Aust. Vet. J.* 2024; 102: 11–18. https://doi.org/10.1111/avj.13293

Ciuchini, F., Adone, R., Pasquali, P., Marianelli, C., Tarantino M., Bandino, E., et al. [Animal Brucellosis: Review of Nonspecific Reactions and Discrepancies Between RBT and CFT]. *Rapporti ISTISAN*, 2005, 05/21.Italian National Institute of Helath. https://www.iss.it/documents/20126/955767/05-21.1129032034.pdf/c4283476-1b3b-3b0a-f429-57aa480fa04c?t=1575578973803

Cloeckaert, A., Zygmunt, M.S., Guilloteau, L.A. *Brucella abortus* vaccine strain RB51 produces low levels of M-like O-antigen. *Vaccine,* 2002. 15;20:1820-2. doi: 10.1016/s0264-410x(02)00035-x. PMID: 11906770. https://pubmed.ncbi.nlm.nih.gov/11906770/

Ermolli, M., Folloni, S., Prospero, A., Bellocchi, G., Querci, M., Petracca, G., et al. (2008). Development of a multiplex immunoassay based on the ELISA Reverse m&d for the simultaneous detection of enzootic bovine leukosis (EBL), brucellosis (B) and paratuberculosis (JD) in bovine bulk milk samples. In: Rapid Methods Europe for Food and Feed Safety and Quality, pag. 101. https://www.researchgate.net/publication/268277178_Development_of_a_multiplex_immunoassay_based_on_the_ELISA_Reverse_md_for_the_simultaneous_detection_of_enzootic_bovine_leukosis_brucellosis_and_paratuberculosis_in_bulk_milk_samples

Ermolli, M., Prospero, A., Balla, B., Querci, M., Mazzeo, A., and Van Den Eede, G. (2006). Development of an innovative immunoassay for CP4EPSPS and Cry1AB genetically modified protein detection and quantification. *Food Additives & Contaminants*, *23*(9), 876–882. https://doi.org/10.1080/02652030600699056

Italian Ministry of Health. Decree of May 2, 2024 – Adoption of the mandatory national programmes for the eradication of brucellosis and tuberculosis in cattle and brucellosis in sheep and goats (24A03318). Official Gazette of the Italian Republic No. 151 of June 29, 2024, pp. 17-20. https://www.gazzettaufficiale.it/eli/gu/2024/06/29/151/sg/pdf; https://www.izsler.it/tbcentro/wp-content/uploads/sites/19/2024/12/interno_IZS_Brucellosi.pdf

Petracca, G., Mazzeo, A., Biagetti, M., Papa, P., Prospero, A., Gaeta, C., Rosato M.P. (2004). ELISA reverse technology to simultaneously detect different sanitary parameters in bulk milk. Atti del LVIII Convegno Nazionale della Società Italiana di Scienze Veterinarie (SISVet), Grado (Italy), 23-25 September 2004, pag. 92-93. https://www.researchgate.net/publication/311083354_ELISA-REVERSE_TECHNOLOGY_PER_IL_CONTROLLO_SIMULTANEO_DI_PIU_PARAMETRI_SANITARI_NEL_LATTE_DI_MASSA

WOAH - World Organisation for Animal Health - Manual of Diagnostic Tests and Vaccines for Terrestrial Animals, thirteenth edition 2024 - Chapter 3.1.4. Brucellosis (Infection with *B. abortus, B. melitensis* and *B. suis*)
https://www.woah.org/fileadmin/Home/eng/Health_standards/tahm/3.01.04_BRUCELLOSIS.pdf
