# Supplementary material for: Brucellosis in cattle and buffalo in southern Italian provinces: trends in presence of territory-specific One Health measures
Source: Front Microbiol. 2025 Jun 6;16:1609336. doi: 10.3389/fmicb.2025.1609336 (PMC12179989; doi:10.3389/fmicb.2025.1609336)
Supplement: Supplementary file 4 [file Data_Sheet_4.pdf]

S4

CAMPANIA REGION
RULES FOR CLUSTER AREAS

In the Campania Region, specific infection cluster areas have been identified based on the following criteria (Campania Region, 2022):

- Territories within a 2 km buffer zone surrounding farms with either:
  - at least two active outbreaks in the past two years, with one recurring within the last five years, or
  - three active outbreaks within the last two years.
- Areas covering more than 50% of a municipality's territory.

These cluster areas are primarily located in the province of Caserta, with a smaller presence in the province of Salerno, and are updated annually based on the evolving epidemiological situation.
Currently, four contiguous municipalities in Caserta Province are entirely designated as cluster areas (Cancello ed Arnone, Castel Volturno, Santa Maria La Fossa, Grazzanise).
At the start of the eradication process, under the new One Health approach, seven municipalities were initially classified as cluster zones (the four listed above plus Carinola, Sparanise, and Francolise).
Enhanced biosecurity and control measures are implemented in these cluster areas, including:

- Mandatory vaccination of buffalo heifers aged 6–12 months (only in fully designated cluster municipalities).
- Electronic identification of cattle and buffaloes located in cluster areas using ruminal boluses;
- Biannual health checks for 100% of animals and establishments located within infection cluster areas.
- Negative animals from outbreak establishments may be sent for slaughter as the final load of the day, under veterinary supervision, and using sealed transport.
- Positive animals must be slaughtered as soon as possible, and in any case, within four days of confirmed infection
- In the absence of a suitable isolation area within a facility to effectively separate positive animals, slaughter must occur within 48 hours of detection.
- Upon detection of an outbreak, lactating animals are counted, and the presence of calves is checked in information systems; calves are excluded from breeding programs due to possible latent infection.
- Where structural or operational conditions of multiple premises within cluster areas do not allow effective separation of animals, these are considered a single epidemiological unit by the Veterinary Service. Consequently, all related establishments are subject to the same sanitary measures and control activities. Several such epidemiological units have been identified in Caserta due to the specific geospatial distribution of buffalo farms.
- All premises in infection cluster areas are subject to on-site inspections by local health authority (ASL) veterinarians to verify biosecurity compliance. Where deficiencies are found, farmers are granted a three-month period to meet the required standards. Non-compliance will lead to the application of penalties and measures as provided by applicable regulations.
- Strict compliance with the control program is mandatory. In cases where access is denied to the Veterinary Service for scheduled activities, law enforcement may be called to assist.

Farmers who are uncooperative or obstruct veterinary health checks will face the following sanctions:

a. Ineligibility for any form of public financial support;
b. Prohibition on animal movements, except to slaughterhouses;
c. Ban on marketing milk and dairy products for human consumption.

- For both brucellosis and tuberculosis, movements between disease-free establishments must occur under veterinary restriction using sealed transport, and only following pre-movement testing (RBT and CFT) conducted within 30 days prior, subject to Veterinary Service authorization.
- Movement of animals from disease-free reproductive premises within infection clusters to or from non-cluster areas is prohibited, except in the case of direct transport to slaughterhouses under veterinary restriction and with sealed vehicles (certain case-by-case exemptions may apply, subject to veterinary assessment).
- All transhumance, mountain grazing, and alpine pasture movements within cluster areas are permitted only to identified, geo-referenced, and registered pastures in the National Livestock Database (Banca Dati Nazionale - BDN). Shared use of pastures is prohibited, and all pastures located within infection clusters must be fenced or otherwise delimited.
- Free-range grazing is prohibited in areas affected by *Mycobacterium tuberculosis* complex and brucellosis.

In other non-disease-free areas of the Campania Region, such as Salerno, additional mandatory measures are in place, including:

- Genotyping of all buffalo animals.
- Verification of biosecurity compliance for all establishments (internal farm biosecurity, external perimeter biosecurity, farm management).
- Annual official diagnostic testing of all bovines and buffaloes aged 12 months or older.
- Additionally, all buffalo establishments must conduct bulk milk ELISA testing every two months, with a two-month interval following serological testing in unvaccinated disease-free establishments.
- For farms practicing pasture grazing, transhumance, or alpine grazing, testing must occur prior to animal movement or pasture access.

In all other areas of the Campania Region, the following control measures apply:

1. Serological testing for *Brucella abortus*, *B. melitensis* and *B. suis* infections (and proper tests for bovine tuberculosis infection) is required for all cattle over 24 months and all sheep/goats over 12 months in farms annually identified as at-risk by the Region.
2. Diagnostic activities are conducted by the competent Local Health Authority (ASL) Veterinary Services.
3. Use of electronic equipment for prophylactic operations in livestock facilities.
4. Implementation of a monitoring system to ensure milk from infected animals is not used for human consumption or is thermally treated if used for animal feed. The ASL may use natural dyes to mark milk for disposal or phosphatase testing for milk destined for calves.
5. Enhanced monitoring via the buffalo supply chain traceability platform to verify consistency between the number of milked animals, milk production, births, calves, and abortions. In cases of discrepancy, a consultation with the farm veterinarian is required.

6. Approval and enforcement of protocols for cleaning and disinfecting the contaminated infected premises or those receiving infected animals.
7. Evaluation and approval of the farm's self-monitoring plan submitted by the operator. All related test results (e.g., milk analysis, vaginal swabs, abortions) must be uploaded to the VETINFO system and verified by the authorities.

Reference

CAMPANIA REGION. Mandatory Eradication Programme for Infectious Diseases in Cattle and Buffalo in Campania (DGRC n. 104/2022)
https://oev.izsmportici.it/dgrc-104-2022-approvazione-del-programma-obbligatorio-di-eradicazione-delle-malattie-infettive-delle-specie-bovina-e-bufalina-in-regione-campania/
