# Supplementary material for: Brucellosis in cattle and buffalo in southern Italian provinces: trends in presence of territory-specific One Health measures
Source: Front Microbiol. 2025 Jun 6;16:1609336. doi: 10.3389/fmicb.2025.1609336 (PMC12179989; doi:10.3389/fmicb.2025.1609336)
Supplement: Supplementary file 5 [file Data_Sheet_5.pdf]

S5

A) ITALIAN MINISTRY OF HEALTH

National Extraordinary Commissioner for the control and eradication of bovine, buffalo, ovine, and caprine brucellosis, and bovine and buffalo tuberculosis
Order No. 1/2025, March 6, 2025
Strengthening Measures for the Eradication of Water Buffalo Brucellosis in the Province of Caserta - Official Gazette, General Series No. 60 of March 13, 2025 (25A01570)
https://www.gazzettaufficiale.it/atto/serie_generale/caricaDettaglioAtto/originario?atto.dataPubblicazioneGazzetta=2025-03-13&atto.codiceRedazionale=25A01570&elenco30giorni=false

The Ordinance establishes intervention groups, referred to as Task Forces, coordinated by the National Extraordinary Commissioner for the Control and Eradication of Bovine, Buffalo, Ovine, and Caprine Brucellosis, and Bovine and Buffalo Tuberculosis. These Task Forces are composed of veterinarians, biologists, statisticians, and technical personnel from the National Reference Centre for Brucellosis (CRNB), the National Reference Centre for Veterinary Epidemiology, Planning, Information, and Risk Analysis (COVEPI), and the Veterinary Epidemiological Observatory (OEVR) of the Campania Region.
Their objective is to conduct an in-depth epidemiological analysis of persistent brucellosis outbreaks in water buffalo farms in the province of Caserta, by carrying out inspections and collecting samples for laboratory analysis. The purpose of these activities is to:

- determine the timeframe during which the infection was introduced into the establishment,
- formulate hypotheses regarding the likely cause and origin of the infection,
- clarify whether and how the infection may have spread to other establishments,
- identify risk factors that facilitate the introduction, spread, and persistence of the infection, through an evaluation of management and biosecurity measures implemented on the premises, supported by laboratory testing.

These extraordinary measures are implemented in parallel with those currently in force in the Campania Region.

B) CAMPANIA REGION

Mandatory Eradication Programme for Infectious Diseases in Cattle and Buffalo in Campania (DGRC n. 104/2022)
https://oev.izsmportici.it/dgrc-104-2022-approvazione-del-programma-obbligatorio-di-eradicazione-delle-malattie-infettive-delle-specie-bovina-e-bufalina-in-regione-campania/

Summary
The eradication of infectious diseases in cattle and buffalo is a fundamental public health objective, ensuring food safety and safeguarding the economic sustainability of livestock farming. The Mandatory Eradication Programme in the Campania region aims to control and eliminate brucellosis, tuberculosis, and other zoonotic diseases affecting bovine and buffalo populations.

Objectives of the Programme

Eradication of Brucellosis and Tuberculosis in cattle and buffalo populations

Prevention of Disease Transmission through strict biosecurity measures
Protection of Public Health by reducing zoonotic risks
Preservation of the Dairy and Meat Industry ensuring product quality and market access
Compliance with European Health Standards to maintain international trade eligibility

Control and Monitoring Measures

Annual Serological Testing of 100% of the bovine and buffalo populations in farms subject to brucellosis and tuberculosis control
Bacteriological and Molecular Testing on aborted fetuses, lochia, vaginal swabs, and milk samples
Identification and Traceability of all livestock through electronic tagging and registration in the National Database (BDN)
Movement Restrictions to prevent the spread of infections, including pre-movement health checks
Biosecurity Audits across all farms to ensure compliance with disease prevention protocols
Mandatory Vaccination in high-risk areas, particularly using the RB51 strain for buffalo populations in cluster municipalities
Sanitary Slaughter and Compensation Measures for infected herds, ensuring financial support for affected farmers

Extraordinary Measures (2022-2027)

Implementation of six-year eradication strategies to declare cluster areas free from brucellosis and tuberculosis
Environmental Risk Management, including control of drainage systems and prevention of flooding in agricultural areas
Structural Enhancements to support farms undergoing repopulation after disease outbreaks

Biosecurity Protocols

External Biosecurity: Farm fencing, controlled access, visitor protocols, and pest control
Internal Biosecurity: Sanitation procedures, quarantine measures, separation of animal categories, and proper effluent management
Water Safety: Mandatory annual microbiological testing of farm water sources

Implementation and Compliance

The programme is enforced by regional veterinary authorities, with periodic inspections and penalties for non-compliance. The Epidemiological Veterinary Regional Observatory (OEVR) oversees the disease monitoring system and updates infection cluster maps every six months.

This mandatory eradication programme is essential for ensuring sustainable livestock farming, protecting human health, and maintaining Campania's reputation as a leading producer of high-quality dairy and meat products.
The extraordinary measures will remain in force for six years, until December 31, 2027, with the ultimate goal of declaring the affected cluster areas free from brucellosis in compliance with EU regulations.

- **Environmental Biosecurity**: Due to the limited maintenance of drainage canals and the insufficient capacity of the Regi Lagni drainage system to channel water to the sea, heavy rainfall often causes severe flooding in agricultural areas and farms, significantly increasing the risk of pathogen spread.

- **Structural Deficiencies and Farm Density**: Support measures are required, particularly for farms seeking to repopulate after culling (stamping-out).
- **Infection Cluster Areas and Movement Restrictions**
- **Cluster Areas**: Defined as territories within a 2 km buffer around establishments with at least two active outbreaks in the past two years (one of which must have recurred in the last five years) or three active outbreaks in the last two years. The buffer is determined considering both natural and artificial barriers such as rivers and watercourses.
- **Cluster Municipalities**: If the infection cluster covers more than 50% of a municipality's territory, the entire municipality is designated as a "cluster municipality".
- **Movement Restrictions**: The movement of livestock from establishments within cluster areas to non-cluster areas is strictly prohibited, except for direct transport to slaughterhouses under veterinary supervision with sealed transport vehicles. Within cluster areas, all livestock movements must occur under veterinary supervision with sealed vehicles, and brucellosis testing (BRC) must be conducted within 30 days before transport.
- **Monitoring and Updates**: The Epidemiological Veterinary Regional Observatory (OEVR) updates the list of infection cluster areas and affected establishments annually.

Brucellosis Vaccination Plan

- Mandatory Vaccination: The RB51 strain vaccine is administered, including a booster dose, to buffaloes aged 6-12 months in cluster municipalities, which account for approximately 84% of outbreaks recorded in the last six years in the province of Caserta.
- Voluntary Vaccination: On a voluntary basis, vaccination with the RB51 strain is also available in buffer municipalities adjacent to cluster territories and in the remaining non-cluster municipalities.
- Regulatory Compliance: The vaccination plan has been approved by the European Commission through the Ministry of Health.

Biosecurity Measures

Biosecurity is the set of management and physical measures applied to prevent the introduction of new diseases and infections into a disease-free population and to limit their spread when present.

1. Environmental Biosecurity:
    - Management of drainage canals.
    - Preventing illegal discharges into drainage channels.
    - In areas at high-prevalence of brucellosis, green fodder is prohibited as animal feed.
2. External Biosecurity:
    - Separation barriers, fencing, and shelters where applicable.
    - Sanitation procedures, disinfection, pest and insect control.
    - Clear signage restricting access to unauthorized personnel.
    - Signage outlining procedures for visitors and movement between risk zones.
    - Farms must not contain drainage canals or watercourses within their perimeter; where such features exist, a containment system must prevent floodwater ingress or the release of contaminated wastewater.
3. Internal Biosecurity:
    - Entry and exit protocols for personnel, animals, and supplies.
    - Equipment usage protocols.
    - Designated areas for different categories of livestock.

- Quarantine measures for newly introduced animals.
- Properly distanced and adequately sized isolation and infirmary facilities.
- At least two separate, regularly maintained and secured storage areas for livestock effluents.
- Secure and potable water supply, with annual microbiological testing for water from wells or cisterns (wells must be deeper than 30 meters and fully lined).
- Compliance with minimum space requirements per animal as defined in Annex B of the Plan.
- Proper facilities for calving, cleaning, and disinfecting equipment and vehicles.
- Dedicated storage for by-products (e.g., dead animals, aborted fetuses, placentas) with timely and appropriate disposal by authorized companies.

Farm Management Guidelines

1. Animal Movement: avoid purchasing livestock from farms with brucellosis or tuberculosis outbreaks within the past three years.
2. Personnel and Visitors: Ensure compliance with biosecurity protocols for staff, suppliers, buyers, transporters, and visitors.
3. Equipment Use: contractors handling livestock waste must follow strict disinfection protocols for vehicles and equipment. Shared use of equipment is strongly discouraged.
4. Effluent Management: Ensure appropriate handling and disposal of livestock waste
5. Hygiene Protocols: maintain rigorous sanitation practices across all farm operations.

Specific Objectives:

1. **Public Health Protection:** To reduce the risk of brucellosis transmission to humans by enhancing food safety and safeguarding public health.
2. **Economic Sustainability:** To improve the economic viability of livestock farms by reducing the costs associated with disease management and increasing overall productivity.

The achievement of the program's objectives is closely linked to addressing the following critical conditioning factors:

- **Environmental Biosecurity and Management of Drainage Channels:** Inadequate maintenance of wastewater disposal channels, combined with the limited capacity of the *Regi Lagni* system to collect and discharge water into the sea, results—particularly during periods of heavy rainfall—in significant flooding of agricultural areas and livestock facilities. This contributes to an increased risk of pathogen dissemination.
- **Structural Deficiencies and High Concentration of Livestock Facilities:** Financial support measures are needed for farms seeking to repopulate following stamping-out procedures.
- **Legal Challenges by Owners of Infected Establishments:** Precautionary suspension orders issued by administrative courts and the Council of State exacerbate the risk of pathogen spread in the environment. These rulings pose a serious threat to both public and animal health, as they hinder Competent Authorities from implementing essential measures to control *Brucella* and *Mycobacterium tuberculosis* complex infections.
